# Lsh/HELLS is required for B lymphocyte development and immunoglobulin class switch recombination

**DOI:** 10.1073/pnas.2004112117

**Published:** 2020-07-29

**Authors:** Yafeng He, Jianke Ren, Xiaoping Xu, Kai Ni, Andrew Schwader, Richard Finney, Can Wang, Lei Sun, Kimberly Klarmann, Jonathan Keller, Anthony Tubbs, Andre Nussenzweig, Kathrin Muegge

**Affiliations:** ^a^Epigenetics Section, Mouse Cancer Genetics Program, Center for Cancer Research, National Cancer Institute, Frederick, MD 21702;; ^b^CCR Collaborative Bioinformatics Resource, Center for Cancer Research, National Cancer Institute, Bethesda, MD 20892;; ^c^Hematopoiesis and Stem Cell Biology Section, Mouse Cancer Genetics Program, Center for Cancer Research, National Cancer Institute, Frederick, MD 21702;; ^d^Basic Science Program, Leidos Biomedical Research, Inc., Basic Science Program, Frederick National Laboratory, Frederick, MD 21702;; ^e^Laboratory of Genome Integrity, National Cancer Institute, NIH, Bethesda, MD 20892

**Keywords:** ICF syndrome, class switch recombination, Lsh, chromatin remodeler

## Abstract

Mutation of HELLS (Helicase, Lymphoid-Specific)/Lsh in human DNA causes a severe immunodeficiency syndrome, but the nature of the defect remains unknown. We assessed here the role of Lsh in hematopoiesis using conditional Lsh knockout mice with expression of Mx1 or Vav Cre-recombinase. Bone marrow transplantation studies revealed that Lsh depletion in hematopoietic stem cells severely reduced B cell numbers and impaired B cell development in a hematopoietic cell-autonomous manner. Lsh-deficient mice without bone marrow transplantation exhibited lower Ig levels in vivo compared to controls despite normal peripheral B cell numbers. Purified B lymphocytes proliferated normally but produced less immunoglobulins in response to in vitro stimulation, indicating a reduced capacity to undergo class switch recombination (CSR). Analysis of germline transcripts, examination of double-stranded breaks using biotin-labeling DNA break assay, and End-seq analysis indicated that the initiation of the recombination process was unscathed. In contrast, digestion–circularization PCR analysis and high-throughput sequencing analyses of CSR junctions and a chromosomal break repair assay indicated an impaired ability of the canonical end-joining pathway in Lsh-deficient B cells. Our data suggest a hematopoietic cell-intrinsic role of Lsh in B cell development and in CSR providing a potential target for immunodeficiency therapy.

## Significance

ICF4 is an inherited disease with early mortality due to immunodeficiency. Although the genetic cause is known to be a mutation in the Lsh (lymphoid-specific helicase) gene, it remains unknown why patients suffer from an impaired immune response. Using conditional Lsh knockout mouse models, we provide evidence that Lsh has a hematopoietic cell-intrinsic role in promoting early B cell development and that deficient B cells fail to conduct the step of DNA end joining that is required to yield a variety of immunoglobulin isotypes during class switch recombination. Our data uncover an essential role of Lsh/HELLS in immunoglobulin production which could lead to improved therapeutic options to treat ICF4 patients.

Lsh (Lymphoid-Specific Helicase) has been identified in lymphocytes and belongs to a family of chromatin remodeling proteins that are related to DNA helicases (1, 2). Lsh modulates the establishment of DNA methylation patterns during cellular differentiation and regulates chromatin accessibility in vitro and in vivo (3–14). Targeted deletion of Lsh in mice is lethal, and Lsh mutant embryos display multiple organ and stem cell defects (15–20). In addition, Lsh has been implicated in tumorigenesis and in the repair of DNA damage (21–23).

Mutation of human Lsh, also known as HELLS (Helicase, Lymphoid-Specific), causes an Immunodeficiency, Centromeric Instability and Facial Anomalies (ICF) 4 syndrome which is characterized by recurrent fatal respiratory and gastrointestinal infections associated with hypogammaglobulinemia and immunodeficiency (24–26). Three other genes are currently known to cause ICF syndrome upon mutation, among them DNA methyltransferase 3B (DNMT3B) (ICF1), mutations in zinc finger and BTB domain containing 24 (ZBTB24) (ICF2), and cell division cycle associated 7 gene (CDCA7) (ICF3).

Despite the discovery of HELLS mutations associated with the ICF syndrome (26), the pathophysiological pathways underlying the disease remain unresolved including the causes of reduced Ig levels (24). Because constitutive deletion of Lsh in mice is lethal (17, 19), we generated conditional knockout (CKO) mice to study Lsh’s role in the hematopoietic system. We report here that Lsh deficiency results in abnormal B lymphocyte development and diminished IgG class switch recombination (CSR). Using high-throughput sequencing, we demonstrate that the defect is not due to a failure to initiate DNA double-stranded breaks but is based on an impaired joining process. Our CKO mice provide insights into the pathophysiology of the ICF disease process which may improve therapeutic options for ICF patients.

## Results

### Impaired B Cell Development upon Lsh Depletion.

**Fig. 1.**
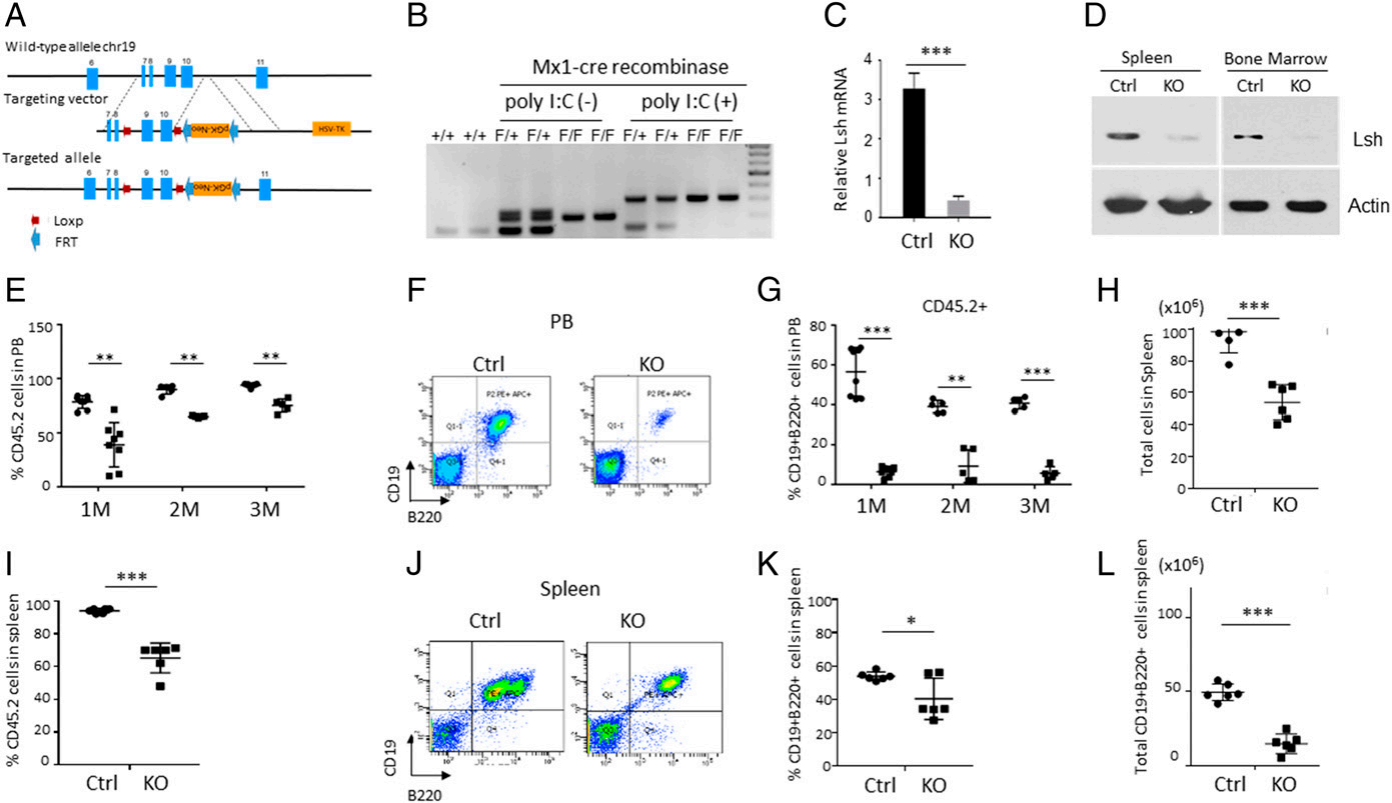
Hematopoietic development in Lsh CKO mice. (*A*) Graph of the targeting strategy. (*B*–*D*) Cre-recombinase was induced by injection of poly (I:C) to generate Lsh KO mice (Mx1^Cre/+^Lsh^fl/fl^ with poly [I:C] administered) and Ctrl mice (Mx1^Cre/+^Lsh^fl/+^ with poly [I:C] administered). Lsh depletion was confirmed by PCR analysis using genomic DNA derived from BM (100% chimera), *n* = 3 (*B*), by RT-PCR analysis using RNA derived from BM (100% chimera), *n* = 3 (*C*), and Western analysis (*D*) 2 wk after injection. (*E*) FACS (Fluorescence-activated cell sorting) analysis for detection of CD45.2^+^ donor cells in the peripheral blood (PB) of irradiated CD45.1^+^ recipient mouse 1, 2, or 3 mo after BMT (100% chimera); Ctrl cells (●) or Lsh KO cells (■). (*F* and *G*) FACS analysis for detection of B cells (CD19^+^B220^+^) among CD45.2^+^ cells in PB. (*H*) Total number of splenocytes. (*I*) Proportion of CD45.2^+^ donor cells in the spleen. (*J* and *K*) FACS analysis for detection of CD19^+^B220^+^ among CD45.2^+^ cells in spleen. (*L*) Total number of CD19^+^B220^+^CD45.2^+^ cells in spleen. **P < 0.01; ***P < 0.001.

To study the effect of Lsh in the hematopoietic system we created a conditional Lsh knockout (KO) mouse that allows tissue-specific deletion of exon9 and exon10 of the Lsh gene (Fig. 1*A* and *SI Appendix*, Fig. S1 *A*–*D*). Exon 9 and 10 comprise an ATP-binding domain and a DEAD box motif that are critical for chromatin remodeling function of Lsh (9). Floxed (Lsh fl/fl) mice, which had been intercrossed with Actin-Cre recombinase transgenic mice, showed widespread deletion of Lsh and displayed early lethality in agreement with previous observations (*SI Appendix*, Fig. S1 *E* and *F*) (17). To delineate the role of Lsh in adult hematopoietic stem cells, we crossed Lsh fl/fl mice to IFN-inducible Mx1-Cre recombinase transgenic mice (Mx1Cre/+) (27). Cre-recombinase was induced by injection of polyinosinic-polycytidylic acid (poly [I:C]), and Lsh depletion was confirmed by genomic DNA PCR, messenger RNA (mRNA), and Western analysis of mouse bone marrow cells (Fig. 1 *B*–*D*). The frequency of Lin- Sca-1+c-Kit+ hematopoietic stem and progenitor cells derived from the bone marrow (BM) of Lsh KO mice (Mx1Cre/+Lsh fl/fl with poly [I:C] administered) was comparable to that of control (Ctrl) mice (*SI Appendix*, Fig. S2 *A* and *B*). To investigate the developmental potential of Lsh KO hematopoietic stem and progenitor cells (HSPCs) in a wild-type host environment, we transplanted 1 × 10^6^ BM cells (CD45.2+) from Lsh KO or Ctrl mice into lethally irradiated recipient mice (CD45.1+) to track donor reconstitution (100% chimeras) by flow cytometry (*SI Appendix*, Fig. S2*C*). The frequency of Lsh KO donor cells in the peripheral blood was significantly reduced at 1, 2, and 3 mo after bone marrow transfer (BMT) compared to Ctrl donor cells (Fig. 1*E*). In particular, the proportion of CD19+B220+ expressing B cells in the peripheral blood was severely decreased (Fig. 1 *F* and *G*), whereas the relative frequency of Mac1+Gr1+ myeloid cells was increased, and the proportions of CD4+ and CD8+ T cells were unchanged (*SI Appendix*, Fig. S3 *A*–*E*). The number and frequency of Lsh KO donor cells in the spleen was significantly lower than Ctrls (Fig. 1 *H* and *I*). Most notably, the proportion of CD19+B220+ B cells was decreased by half, and the total number of CD19+B220+ B cells was significantly reduced about fourfold compared to Ctrls (Fig. 1 *J*–*L*). The thymus showed a decrease in the frequency and total number of the CD4+ subset, whereas CD4+CD8+ or CD8+ subsets were unchanged (*SI Appendix*, Fig. S3 *J*–*Q*). The spleen displayed a decrease in relative frequency of CD4+ T cells and absolute numbers of CD4+ and CD8+ T cells in the absence of Lsh (*SI Appendix*, Fig. S3 *F*–*I*).

In addition, we used competitive transplants to further evaluate the ability to produce differentiated cells of Lsh-deficient HSPCs compared to wild-type HSPCs and to provide an internal technical control for the efficacy of irradiation and injection. We mixed BM cells (CD45.2+) from Lsh KO (or Lsh Ctrl) mice with BM cells from wild-type mice (CD45.1+) at equal proportions (50:50 chimeras) and injected them into lethally irradiated recipient mice (CD45.1+). We observed a significant decrease in the frequency of CD19+B220+ expressing B cells in the peripheral blood, whereas Mac1+Gr1+ myeloid cells were relatively increased, and the proportions of CD4+ and CD8+ T cells were unchanged, similar to the results from the noncompetitive BMT (*SI Appendix*, Fig. S4 *A*–*E*). Notably, the frequency of CD19+B220+ expressing B cells in the spleen was reduced, and the total number reduced more than sixfold in Lsh KO mice compared to Ctrl (*SI Appendix*, Fig. S4 *F*–*H*). Furthermore, the bone marrow showed a fivefold reduction in the frequency and total number of CD19+B220+ expressing B cells in Lsh-deficient recipients compared to Ctrls (*SI Appendix*, Fig. S4 *N*–*P*). The total numbers of CD4+ and CD8+ T cells in the spleen was reduced as we had observed in the noncompetitive BMT. The frequency of T cells in the thymus was not significantly altered in Lsh KO recipients (*SI Appendix*, Fig. S4 *I*–*L* and *Q*–*U*). To further evaluate the growth and viability properties of Lsh-deficient B cells in vitro, we purified B cells from the spleen after BMT (*SI Appendix*, Fig. S5*A*) and assessed incorporation of EdU to measure growth and 7AAD staining for viability, as well as propidium iodide and caspase activation for cell death and apoptosis. Lsh-deficient B cells showed a trend of reduced proliferation in response to mitogen in vitro, but it was not significant (*SI Appendix*, Fig. S5 *B* and *C*). Although they displayed increased signs of cell death and apoptosis, it was only marginal (*SI Appendix*, Fig. S5 *D*–*F*) (*SI Appendix*, Fig. S5 *B*–*F*), suggesting that the in vitro survival ability was only very slightly compromised.

Taken together, Lsh KO HSPCs exhibited significantly reduced reconstitution potential and impaired ability for B lymphocyte development in noncompetitive and competitive bone marrow transplantation assays.

**Fig. 2.**
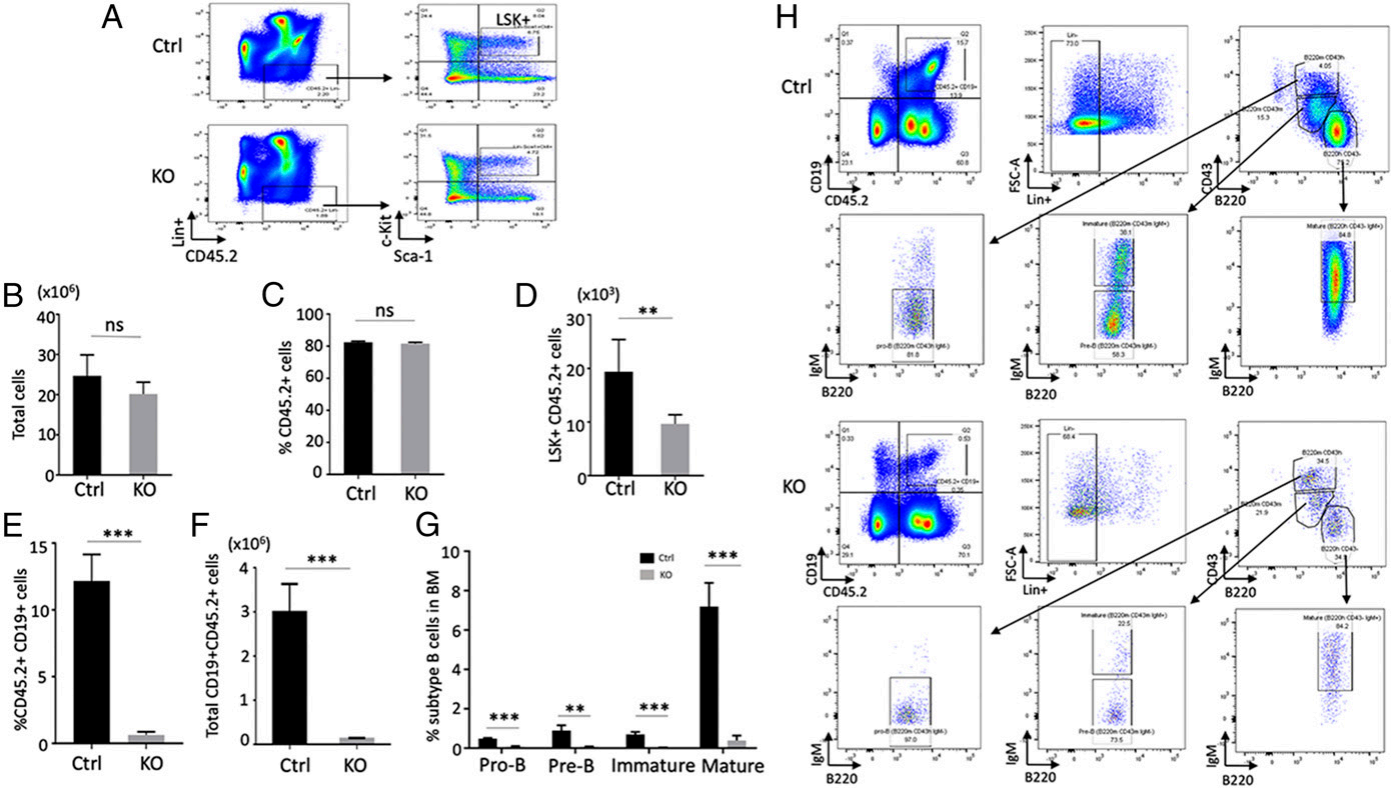
Lsh is essential for B cell development. Characterization of HSPCs (*A*–*D*) and B cell (*E*–*H*) development in the bone marrow of recipient mice 4 mo after BMT (100% chimera). (*A*) FACS analysis for determination of c-Kit^+^Sca1^+^ hematopoietic stem cells from Lin-CD45.2^+^ donor cells in Lsh KO and Ctrl BM. (*B*–*D*) Total BM cells (*B*), proportion of CD45.2^+^ donor cells (*C*), and Lin^−^Sca1^+^c-Kit^+^ (LSK^+^) hematopoietic stem cells (*D*) after BMT; *n* = 5 per group. (*E* and *F*) Proportion of CD19^+^ cells (*E*) among CD45.2^+^ donor cells in the BM and total number of CD19^+^CD45.2^+^ B cells in BM. (*G* and *H*) Frequency of pro-B cell, pre-B cell, immature and mature B cells among CD45.2^+^ donor cells in Lsh KO and Ctrl BM; *n* = 5 per group. (*H*) Representative flow cytometry profiles of pro-B cell, pre-B cell, immature and mature B cells among CD45.2^+^ donor cells, as determined by cell surface markers CD19, CD43, B220, and IgM. *n* = 5 per group. *P < 0.05; **P < 0.01; ***P < 0.001; ns, not significant.

To further investigate the role of Lsh in B cell development, we assessed the cellular composition in the BM of recipient mice after BMT (100% chimeras) (28). The subset of Lin- Sca-1+c-Kit+ HSPCs was reduced by half in mice transplanted with Lsh KO BM compared to Ctrl BM (Fig. 2 *A*–*D*), whereas the total number of BM cells and the number of engrafted (CD45.2) donor cells was unaltered (Fig. 2 *B* and *C*). The frequency of CD19+ B cells among CD45.2+ donor cells was more than 10-fold reduced in mice KO BM compared to Ctrl BM, and total B cell numbers were significantly decreased, confirming that Lsh is intrinsically required in HSPCs for normal B cell output of the BM (Fig. 2 *E* and *F*). The frequency and number of Pro-B, Pre-B, immature and mature B cells were all reduced in KO BM and only 10–15% compared to that in Ctrl BM (Fig. 2 *G* and *H*), suggesting that Lsh is required at early stages of B cell development. Consistent with the observation of reduced numbers of mature B cells, serum Ig levels were greatly diminished in KO recipients compared to Ctrl recipients at 1, 2, and 3 mo after BMT (*SI Appendix*, Fig. S6).

Our data suggest that impaired hematopoiesis was caused by hematopoietic cell-autonomous effects of Lsh deletion, rather than indirect effects via the BM microenvironment and that Lsh is required from the early stages of B cell development. The decrease in mature B cell numbers may contribute to the shortage of immunoglobulins we had observed in KO BMT recipients.

### Reduced Ig Production in the Absence of Lsh.

**Fig. 3.**
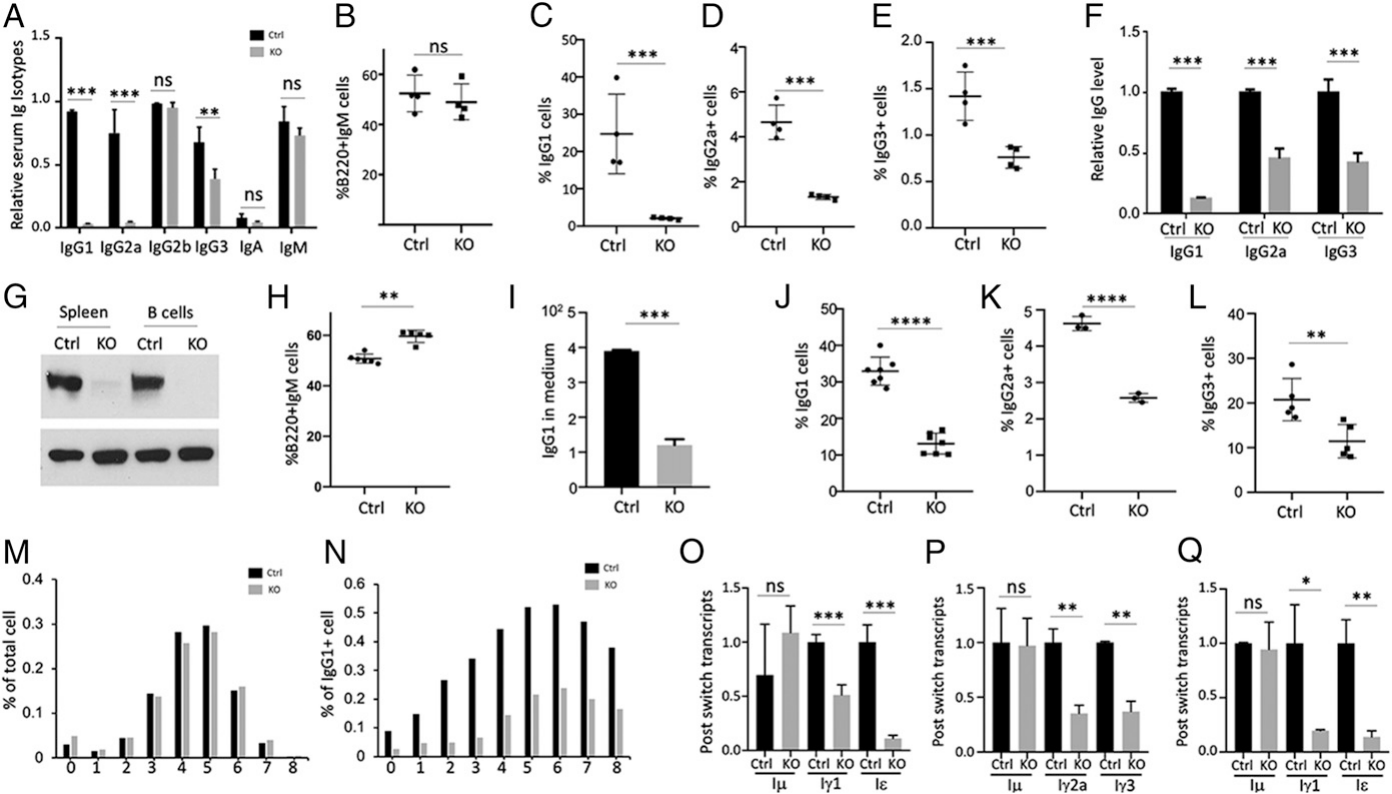
Impaired in vivo and in vitro Ig secretion in Lsh-depleted Mx1-cre and Vav-cre mice. (*A*) ELISA analysis of serum Ig isotypes from Mx1^Cre/+^Lsh^fl/fl^ (KO) and Mx1^Cre/+^Lsh^fl/+^ (Ctrl) mice 2 wk after poly (I:C) injection, *n* = 8 per group. (*B*) FACS analysis of B220^+^IgM^+^ B cells in KO and Ctrl spleen. (*C*–*E*) Surface expression of Ig isotypes IgG1 (*C*), IgG2a (*D*), and IgG3 (*E*) on CD19^+^ purified KO and Ctrl splenocytes was determined by FACS analysis 3 d after stimulation with LPS and IL-4 or IFNγ, *n* = 4; (*F*) ELISA of Ig isotypes in culture supernatants. (*G*) Western analysis for detection of Lsh protein in Lsh Vav KO (Vav^Cre/+^Lsh^fl/fl^) compared to Ctrl mice (Vav^Cre/+^Lsh^fl/+^). (*H*) FACS analysis for detection of B220^+^IgM^+^ B cells in Lsh Vav KO and Ctrl spleen. (*I*) ELISA of IgG1 release after 3 d culture of purified CD19^+^ Lsh Vav KO and Ctrl splenocytes. (*J*–*L*) Surface expression of indicated Ig isotypes in Lsh Vav KO and Ctrl CD19+ splenocytes stimulated in vitro. (*M* and *N*) CFSE-pulsed purified B cells derived from Lsh Mx1 KO (KO) or Ctrl (Ctrl) mice without BMT after 4 d of stimulation with LPS plus IL-4 and FACS analysis for gating populations based on the intensity of CFSE which represents the number of cells in each generation and the percentage of IgG1 expressed cells in each generation. (*O*–*Q*) RT-qPCR analysis for detection of IgM or postswitch transcripts for IgG1 and IgE (*O*) and IgG2a and IgG3 (*P*) in purified B cells from Lsh Vav KO (KO) and Ctrl (Ctrl) after 4 d of stimulation with LPS plus IL-4 (*O*) or LPS plus IFNγ (*P*), or for detection of IgG1 and IgE (*Q*) in purified B cells from Lsh Mx1 KO (KO) and Ctrl (Ctrl) mice without BMT after 4 d of stimulation with LPS plus IL-4. *P < 0.05; **P < 0.01; ***P < 0.001; ****P < 0.0001; ns, not significant.

ICF4 patients display reduced Ig levels in the presence of normal lymphocyte numbers and no apparent in vitro proliferation defect in response to mitogens (29, 30). We noted that Lsh KO mice that had not received BMT displayed reduced serum levels of IgG1, IgG2a, and IgG3 compared to Ctrls (Fig. 3*A*). This phenotype was stable up to 12 and 20 wk after Lsh deletion and did not show recovery (*SI Appendix*, Fig. S7 *A* and *B*). Since the proportions and absolute numbers of B cells and other lineages in the bone marrow, blood, or in peripheral lymphoid organs were unaltered in mice without BMT (*SI Appendix*, Fig. S7 *C*–*F*), we concluded that the lack of immunoglobulins was not simply due to an absence of B cells or decrease in the numbers of costimulatory T cells. In addition, we found no significant changes in serum levels of IL-2, IFNγ, IL-4, IL-6, IL-1β, and IL-10 comparing Lsh KO mice to Ctrls (*SI Appendix*, Fig. S8 *A*–*F*). This suggested that lessened Ig levels occurred independent of reduced B cell numbers, skewed CD4+ T cell proportion or abnormal cytokine secretion.

We hypothesized that the lack of immunoglobulins was caused by a B cell defect and reflected a reduced capacity of Ig production. To this end, we tested whether Lsh-depleted purified B cells would be able to generate Ig isotypes in vitro in response to appropriate stimulation. Resting naive B cells express IgM but undergo CSR upon stimulation and express Ig isotypes (31). KO spleen displayed a similar proportion of IgM+B220+ B cells compared to Ctrl (Fig. 3*B*). After appropriate stimulation with LPS (Lipopolysaccharides) and IL(Interleukin)4 or IFN(Interferon)γ, purified KO CD19+ B cells displayed only 2% IgG1 on their surface compared to 25% on Ctrl cells (Fig. 3*C*). Likewise, IgG2a and IgG3 surface expression were reduced to 28% and 54% in Lsh KO cells compared to Ctrls (Fig. 3 *D* and *E*). Consistent with the loss of surface Ig expression, Lsh KO cultures released significantly less IgG1, IgG2a, and IgG3 into the supernatant compared to Ctrls (Fig. 3*F*).

To corroborate these findings, we employed a second mouse model using Vav-cre recombinase transgenic mice in which the Lsh gene is deleted in definitive hematopoietic stem cells beginning in the fetal liver (32). We confirmed decreased Lsh protein expression in the spleen and in purified B cells derived from Lsh Vav KO (VavCre/+Lsh fl/fl) compared to Ctrl mice (VavCre/+Lsh fl/fl) (Fig. 3*G*). As in the Mx1 model, the number of IgM+B220+ B cells was not reduced in Lsh Vav KO spleen compared to Ctrls (Fig. 3*H*). Yet, in vitro stimulation for induction of isotypes revealed lower levels of Ig isotype surface expression and isotype secretion in Lsh Vav KO cultures compared with Ctrls (Fig. 3 *I*–*L*).

Next, we determined whether the deficiency in CSR was due to altered cell proliferation or a decrease in survival. First, we examined proliferation which is required for successful recombination (31). Overall, cell numbers and EdU incorporation (to measure replication) were indistinguishable comparing Lsh KO cells (without BMT) to Ctrl cells, suggesting a normal capacity to grow in vitro (*SI Appendix*, Fig. S9 *A*–*C*). Using cytoplasmic dye dilution (CFSE) in combination with flow cytometric staining we examined surface Ig isotype expression of purified KO B cells (without BMT) after several days of in vitro stimulation to determine clonal expansion of switched and nonswitched cells. The profile of CFSE staining was similar in Lsh-depleted cells compared to Ctrl cells, reflecting an equivalent proportion of cell divisions in both cultures (Fig. 3*M*). Surface expression of IgG1 increased in both samples with subsequent number of cell divisions (Fig. 3*N*). The percentage of IgG1+ Lsh KO B cells was similarly reduced compared to Ctrl cells with an identical number of cell divisions. These results suggest that a defect in Ig generation is independent of differential clonal expansion. In addition, we examined cell death in Lsh KO B cells (without BMT); however, neither staining for propidium iodide, measuring viability and death, nor annexin V to detect early apoptosis or caspase to detect late apoptosis displayed any significant differences comparing Lsh-deficient cells to Ctrls (*SI Appendix*, Fig. S9 *D* and *E*). We conclude that the failure to generate immunoglobulins could not be simply explained by compromised cell growth or survival.

### Reduced Postswitch Transcripts, Normal AID Levels, Germline Transcripts, and Generation of DSBs in the Absence of Lsh.

To delineate the nature of the Ig deficiency, we directly assessed the generation of postswitch transcripts and found a decrease for IgG1, IgE, IgG3, and Ig2a for postswitch transcripts in purified CD19+ B cells derived from Lsh Vav KO mice compared to Ctrl (Fig. 3 *O* and *P*). Similar results were observed in Lsh Mx1 KO B cells compared to their respective Ctrls (Fig. 3*Q*). Thus, a decline in transcript levels was responsible for the Ig deficiency in the absence of Lsh.

**Fig. 4.**
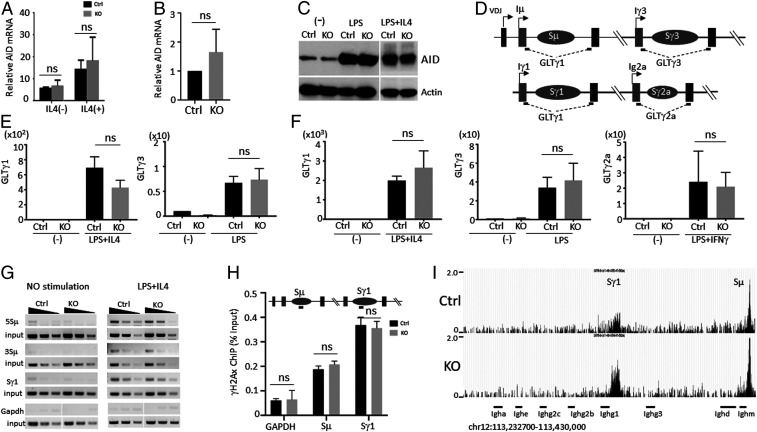
Analysis of chromatin accessibility and CSR initiation. (*A* and *B*) RT-PCR analysis for detection of AID mRNA expression in Lsh Mx1 KO and Ctrl purified B cells after stimulation with LPS with or without IL4 (*A*), or Lsh Vav KO and Ctrl B cells (*B*) stimulated with LPS. (*C*) Western blot analysis for detection of AID protein in B cells from Lsh Vav KO and Ctrl mice, after stimulation with LPS or LPS and IL-4 for 4 d. (*D*) Graph of IgH locus indicating position of primers for detection of germline transcripts. (*E* and *F*) qPCR analysis for detection of germline transcripts for IgG1, IgG3, and IgG2a in CD19+ B cells from Lsh Mx1 KO (*E*) or Lsh Vav KO (*F*) and their respective Ctrls stimulated for 24 h to undergo CSR. (*G*) Biotin-labeling DNA break assay was used for detection of DSBs in the IgM (Sμ) and IgG1 (Sγ1) switch regions after 2 d of LPS/IL4 activation of Lsh Vav KO and Ctrl purified B cells. Samples were threefold serially diluted. Gapdh served as negative Ctrl. (*H*) Chromatin immunoprecipitation analysis for detection of γH2Ax in switch regions Sµ and Sγ. Amplicon locations are indicated in scheme as a black line. (*I*) University of California, Santa Cruz genome browser view illustrating the distribution of DSBs (normalized read density as reads per million) detected by End-seq analysis 2 d after stimulation with LPS/IL4/anti-CD180 of Lsh Vav KO and Ctrl purified B cells. *P < 0.05; ***P < 0.001; ****P < 0.0001; ns, not significant.

Next, we investigated whether the deficiency in Ig production was due to a problem with the initiation of CSR. To this end, we first evaluated the expression of the activation-induced deaminase (AID) gene, which is essential for onset of Ig CSR (33). The amount of AID m(messenger)RNA and protein was not different in Lsh Mx1 KO or Vav KO purified B cells compared to Ctrl cells (Fig. 4 *A*–*C*), suggesting that AID expression was not a limiting factor. The induction of germline transcripts (Fig. 4*D*), which serve as indicator for chromatin accessibility in switch regions and which are required for initiation of CSR by AID (34), was not altered in Lsh Mx1 or Vav KO B cells compared to their respective Ctrls (Fig. 4 *E* and *F*). This suggested that Lsh deficiency did not alter chromatin accessibility at switch regions with respect to transcription.

To assess whether CSR can be initiated in the absence of Lsh, we analyzed the occurrence of DNA double-stranded breaks (DSBs) in switch regions. AID deaminates deoxycytosines in switch region DNA, yielding deoxyuracils (31, 35). The processing results in the introduction of DSBs in the upstream donor and downstream acceptor switch regions. Purified B cells from Lsh Vav KO mice and Ctrls were stimulated for switch recombination, and DSBs were end-labeled with biotinylated dUTP (36). We found equal amounts of DSBs in the Sμ and Sγ1 switch regions in both samples, suggesting a similar capacity to induce breaks in the presence or absence of Lsh (Fig. 4*G*). The DSBs marker γH2AX, which is critical for successful switch recombination (35), was present at comparable levels at switch regions comparing Lsh KO B cells with Ctrl B cells (Fig. 4*H*). To use an unbiased approach to quantitatively determine initiation of DSBs during switch recombination, we performed End-seq analysis (37). Lsh KO B cells and Ctrl B cells were induced to switch recombination for 48 h, and DSBs were captured with streptavidin-coated beads and detected by high-throughput sequencing. The number of reads mapping to the genome are proportional to the frequency of DNA ends in the cell population (37). The frequency of DSBs in Sμ and Sγ1 switch regions was not reduced in Lsh KO samples but rather increased compared to Ctrls (Fig. 4*I* and *SI Appendix*, Fig. S10*A* and Table S2).

Our data indicate that Lsh-deficient cells are capable of DNA cleavage in switch regions and that the defect in switch recombination is downstream of chromatin accessibility and the formation of DSBs.

### Lsh Deficiency Impairs Ig Class Switching at the Level of Recombination.

**Fig. 5.**
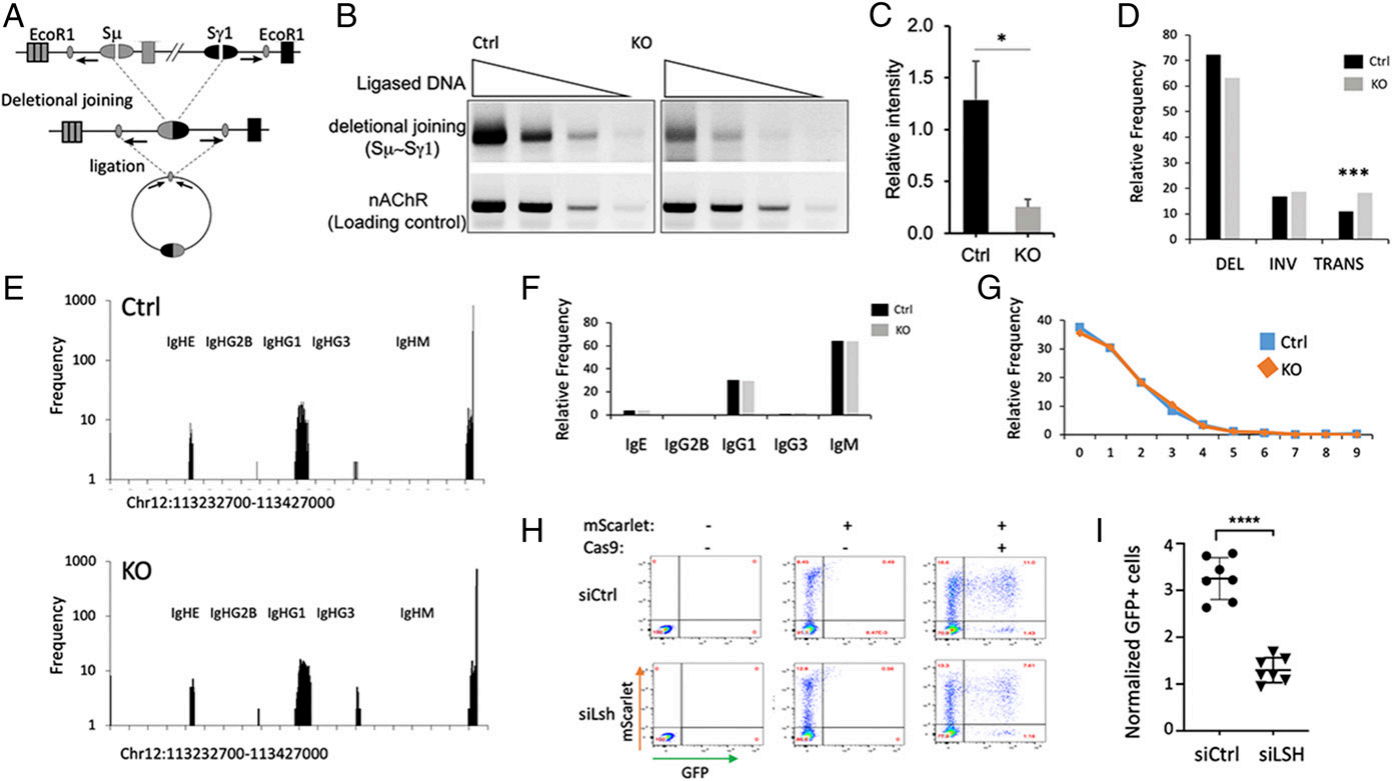
Junction and end-joining analysis. (*A*) Schematic representation of DC–PCR assay. (*B*) DC–PCR results from LPS/IL-4–activated of Lsh Vav KO and Ctrl purified B cells. Amplification of the nAChR locus that does not undergo recombination served as Ctrl for DNA input, efficiency of digestion and circularization. (*C*) Gel band quantification and summary of DC–PCR results including results from *SI Appendix*, Fig. S10. (*D*) HTGTS analysis of junctions derived from LPS/IL4-activated Lsh Vav KO and Ctrl purified B cells to discern deletion (DEL), Inversion (INV), and Translocation (TRANS). χ^2^ statistic, *P* value is <0.00001. (*E*) HTGTS analysis to determine chromosom al distribution of junctions on chr12. (*F*) Quantification of distribution analysis expressed as percentage. (*G*) Microhomology analysis of Sμ-Sγ junctions in Lsh Vav KO and Ctrl purified B cells after 4 d of in vitro stimulation. More than four mice were analyzed per group. (*H* and *I*) U2OS GFP reporter cells were first treated with si(small interfering)Lsh (targeting Lsh) or siCtrl (nontargeting siRNA) for 48 h and then transfected with expression plasmids for Cas9 and guiding RNAs and control expression plasmids and analyzed by flow cytometry after 48 h. *n* = 6 biologic replicates for each group. *P < 0.05; ***P < 0.001; ****P < 0.0001.

To determine directly whether the defect in Ig production is at the level of recombination, we assessed the capacity to generate switch junctions. We first employed digestion–circularization (DC) PCR analysis, a sensitive assay to quantify junctions generated during deletional rearrangement between Sμ and Sγ1 (Fig. 5*A*) (38). EcoRI-digested genomic DNA was ligated under conditions that favor circularization. Only a deletional rearrangement, but not an unswitched locus, leads to successful amplification on a circularized template (38). The frequency of Sμ–Sγ1 switch junctions in Lsh Vav KO B cells was at least fivefold lower than in Ctrl cells (Fig. 5 *B* and *C* and *SI Appendix*, Fig. S10*B*). This suggests that impaired Ig production reflects a defect at the level of joining recombination intermediates.

To further assess the ability of switch recombination and to gain insights into potential mechanistic defects in the joining process, we sequenced Sµ-Sϒ1 and Sμ-Sε junctions using high-throughput, genome-wide translocation sequencing (HTGTS) (39). After the formation of DSBs, donor and acceptor switch regions are joined by nonhomologous end-joining (NHEJ) (31). When critical factors of the NHEJ pathway are lacking, an alternative joining process is used that favors microhomology (39, 40). Purified Lsh Vav KO B cells and their respective Ctrls were activated for 3 d with LPS and IL4 and recombination junctions recovered through HTGTS. The relative frequency of deletion versus inversions on chromosome 12 and junctions between chromosome 12 and other chromosomes was statistically significantly different with a slight raise of junctions between the Ig locus and other chromosomes in KO samples compared to Ctrls (Fig. 5*D*). The distribution of junctions within the Ig region was similar comparing KO to Ctrl samples (Fig. 5*E*), with the highest frequency of junctions in the Sµ switch region (63%) followed by the Sϒ1 region (30%) and the remainder in Sε and other regions (Fig. 5*F*). About 35% showed “direct” joins consistent with previously published results (39), and the relative incidence of microhomology usage was unaltered in KO samples compared to Ctrls (Fig. 5*G*), suggesting that there was no increase in alternative joining process in the absence of Lsh function (40).

### Reduced End-Joining Capability in Lsh-Deficient Cells.

To directly assess the capacity of performing the canonical NHEJ in Lsh-deficient cells, we employed a recently developed chromosomal DSB reporter assay that is specific for canonical NHEJ (41). After introduction of Cas9-induced breaks, which comprise predominantly blunt end DSBs, a successful chromosomal repair can be detected by green fluorescent protein (GFP)-based reporter. Human siLsh-treated U2OS cells were examined for GFP expression 2 d after transfection with Cas9 and guiding RNA and compared to siCtrl-treated cells. We normalized the frequency of GFP+ cells to transfection efficiency using parallel transfections with a Scarlet or DsRed expression vector. We observed a significant reduction of GFP expression in Lsh knockdown cells (Fig. 5 *H* and *I*). In addition, we measured the efficiency of transfection directly using Cas9 antibodies and found a similar reduction of GFP expression in Lsh-depleted cells (*SI Appendix*, Fig. S11 *A*–*D*). Thus, Lsh depletion diminishes the ability to perform end joining efficiently which impairs CSR proficiency and leads to immunodeficiency.

## Discussion

ICF is a rare and recessive autosomal disorder, and the first documented case was reported several decades ago (42). Although gene mutations have been identified as the cause of the ICF syndrome, the molecular pathways leading to its clinical manifestation have not yet been unraveled. We report here a cell-autonomous effect of Lsh in blood cell development and a critical role for Lsh in B cell maturation. While IgM expressing B cells can evolve at normal frequency, isotype generation is compromised in the absence of Lsh. Our molecular studies reveal an impairment at the level of CSR, and Lsh-deficient B cells show a reduced capacity to form junctions using the nonhomologous end-joining pathway.

Since the mutation in ICF patients occurs in all tissues and cell lineages, it was unclear if the hematopoietic phenotype is due to intrinsic or extrinsic defects such as the bone marrow microenvironment, the vasculature, or hormones (43, 44). Our results point to a hematopoietic cell-autonomous defect in the absence of Lsh. This has implications for future treatment of ICF patients, since, for example, genome editing of stem cells becomes a potential therapeutic strategy in the treatment of ICF4 patients (45). Many factors regulate hematopoiesis, including IL-7 which impairs B cell development (46, 47). However, a defect in IL-7 production cannot account for our findings, since IL-7 is predominantly produced by cells which are not of hematopoietic origin (46, 47), and our results demonstrate a hematopoietic cell-intrinsic defect. While we do not have proof that Lsh has a B cell intrinsic effect, it is a possibility, and reduced IL-7 receptor alpha expression or impaired Stat5 activation may contribute to the phenotype (46, 47). Several changes in chromatin configuration must occur during B cell activation (48). Since Lsh function influences chromatin accessibility and transcription factor binding at enhancer sites (10, 13), it is possible that Lsh may promote B development in part via its epigenetic effects.

Interestingly, the depletion of B cells and skewed T cells was only detected after hematopoietic reconstitution. T cells in the spleen displayed reduced numbers while thymus subsets were unaltered, suggesting a peripheral mechanism which may involve a weakened responsiveness to MHC interactions, cytokine or chemokine signaling, leading to reduced survival of the peripheral T cell pool (49, 50). The results of transplantation assays showed reduced numbers of Lsh-deficient B cells in peripheral blood, spleen, and bone marrow and a marginal decrease of the survival of Lsh-deficient B cells in vitro. An exhaustion phenotype due to proliferative stress can become apparent after transplantation and is sometimes not found in the mutant mice themselves (51). Since donor cells that have been injected in lethally irradiated mice expand rapidly more than 200-fold (52), it is conceivable that replicative stress associated with BMT may affect cellular fitness of Lsh-deficient cells in vivo. Genomic instability is a hallmark of the ICF syndrome (24, 25), and DNA damage may accumulate over time through many cycles of replication and contribute to the reduction of lymphocyte numbers after BMT.

The ICF syndrome shows great clinical variability (24, 25), and some but not all ICF1/ICF2 patient lymphocytes show reduced proliferation in culture (53, 54). Reduced growth and increases in the DNA damage marker gammaH2AX were found in an ICF4 patient-derived cell line and a Lsh KO cell line (55). However, we do not observe significant increases in cell death/apoptosis or reduced growth in Lsh-deficient lymphocytes (without BMT) in short-term in vitro cultures, and a report using primary lymphocytes derived from an ICF4 patient found normal proliferation in response to mitogens and is consistent with our results (30). Furthermore, ICF4 patients have normal peripheral B and T cell numbers (29, 30), consistent with our observation in Lsh-deficient mice that are not undergoing bone marrow reconstitution. The reported differences in cellular survival may be due to the use of an aneuploid transformed cell line (55, 56) versus primary cells (this study and Kubota et al. (30)), culture conditions, the use of IL-4 which promotes B cell survival (57, 58), the duration of cell culture since prolonged culture can perturb DNA methylation patterns in lymphoid cell lines including satellites (59), or the age of the subject from which ICF cells are derived (we used young mice). As mentioned above, a small degree of genomic instability can accumulate over time and may lead to increases in cell death after many cycles of expansion in recurrent immune responses. This can explain the observation that young ICF patients show generally normal numbers of peripheral lymphocytes, whereas older patients have reduced lymphocyte numbers (53, 60, 61). However, other explanations are possible, and more research to understand the nature of reduced B cell numbers in some ICF patients will be interesting in the future. It should be also noted that the heterogeneity of phenotypes among ICF patients in general and among ICF4 patients may be expected since they suffer from diverse genetic mutations that may lead to deletion, malfunction, or negative dominant forms of the protein (24–26, 53, 54, 60–62). Only a few ICF4 patients and HELLS mutation patterns have been reported so far, but other HELLS mutations in ICF4 patients may be described in the future that may cause a defect in B cell numbers as observed in the BMT mice model.

The main symptom of ICF patients is their lack of Ig isotypes (24–26). The generation of isotypes depends on switch recombination, a process that involves breaking and rejoining highly repetitive switch regions in the IgH locus. Our data indicate that the initiation of switch recombination, including induction of AID, generation of accessibility at switch regions and germline transcripts, and formation of DSBs, does not require Lsh. Instead, we provide evidence that the completion of recombination, namely, the joining, is compromised in the absence of Lsh. Since the balance of canonical and alternate end joining is undisturbed as measured by high-throughput sequencing, both pathways appear impacted by Lsh deletion. A recent study reported reduced NHEJ activity in Lsh-deficient cells using a reporter system which requires NHEJ for repair of DSBs induced by SceI expression (55). We extend and confirm their observations using Cas9-induced NHEJ repair and controlling transfection efficiency using a coexpression vector or measuring Cas9 expression. The previous study suggested an involvement of Ku70/80 in the Lsh-mediated repair pathway, but our high-throughput sequencing approach did not reveal a shift to microhomology usage as has been reported in Ku70- or Ku80-deficient B cells (39). In addition, Ku-deficient mice lack B cells since Ku proteins are critical for V(D)J rearrangement, and switch recombination can only be studied after complementation with Ig heavy chain transgenes (63). In contrast, the current study shows normal numbers of B and T cells in adult animals without BMT. More than a dozen molecules are involved in CSR (31, 64), including those involved in the DNA damage response, components of the NHEJ pathway, and factors that induce topological changes in the nuclear architecture (48), and it is possible that Lsh promotes NHEJ activity by modulating known pathways. Interestingly, CSR of IgG2b was not significantly affected in Lsh-deficient B cells. This is not a unique phenomenon, since it was previously reported that individual constant regions can be affected to a variable degree or may be not affected at all during CSR upon depletion of factors influencing NHEJ ability and CSR efficiency (65–67). We can only speculate about the basis for this difference with respect to Lsh’s effect on CSR, but the spatial organization of the Ig locus, the process of loop extrusion, and the stability of the complex that brings broken ends together play a role in CSR (31, 48, 68). It is possible that Lsh’s effect on the chromatin environment is regionally diverse and that this may affect the formation and stability of long-range synapses of different switch regions distinctively. It is also plausible that local changes in the chromatin environment may affect the recruitment signal for end-joining factors diversely, or a locally altered chromatin structure may affect their accumulation differently. While the ability of end joining is reduced at our chromosomal DSB reporter assay, local Lsh effects on the chromatin environment in the Ig locus may exacerbate or ameliorate the detrimental effect on CSR at distinct switch regions differently. It will be interesting to investigate in future studies if and how Lsh affects the chromatin environment in a way that is favorable for successful resolution of DSBs during CSR.

## Materials and Methods

### Generation of Conditional Lsh KO Mouse Strains.

The targeting vector was constructed by cloning 5 kb upstream homologous region including exon7-10 and 2 kb homologous sequence downstream of exon 10. The construct was modified to add two loxP sites 5′ of exon 9 and 3′ of exon 10, a reversed FRT-PGK-Neo-FRT cassette, and a Herpes Simplex Virus thymidine kinase element as a negative selection marker (Fig. 1*A*). The targeting vector was electroporated into 129iTL1 embryonic stem cells (ESCs), and targeted ESC clones were screened for correct homologous recombination by long-range PCR analysis (*SI Appendix*, Fig. S1 *A*–*D*). Mutant ESC clones were injected into C57/B6 carrier blastocysts and transplanted into foster CD1 mothers. Chimeric offspring was bred with wild-type C57BL/6 mice (Charles River Laboratories) to generate Lsh flox/+ mice and with actin-Flp transgenic mice (The Jackson Laboratory) to delete the neomycin cassette flanked by Frt sites. Detailed descriptions of the experiments and computational analyses are provided in *SI Appendix*. Animal care was provided in accordance with the procedures outlined in the “Guide for Care and Use of Laboratory Animals” (69).

## Supplementary Material

Supplementary File

## Data Availability

All relevant data are included in the main manuscript and the *SI Appendix*. High-throughput sequence analysis is available under Gene Expression Omnibus submission GSE150423.
