# Supplementary material for: Lsh/HELLS is required for B lymphocyte development and immunoglobulin class switch recombination
Source: Proc Natl Acad Sci U S A. 2020 Jul 29;117(33):20100–8. doi: 10.1073/pnas.2004112117 (PMC7443918; doi:10.1073/pnas.2004112117)
Supplement: Supplementary File [file pnas.2004112117.sapp.pdf]

Supplemental Information

1. Material and Methods
2. Table S1
3. Table S2
4. Supplemental Figure Legends
5. Supplemental Figures

www.pnas.org/cgi/doi/10.1073/pnas.2004112117

1. Material and Methods

Construction of the targeting vector

To generate conditional knockout alleles of Lsh, the targeting vector was constructed by cloning 5 kb upstream homologous region including exon7-10 and 2 kb homologous sequence at downstream of exon 10. The construct was modified to add two loxP sites at upstream of exon 9 and downstream of exon 10 followed by a reversed FRT-PGK-Neo-FRT cassette using a series of conventional and recombination-mediated cloning methods. The resulting plasmid contains an HSV-thymidine kinase (Tk) element as a negative selection marker (Fig 1A). The Lsh conditional knockout targeting vector sequence was verified by conventional DNA sequencing.

Generation of conditional Lsh knockout mouse strains

To generate conditional Lsh knockout mice, the targeting vector was electroporated into 129iTL1 embryonic stem cells (ESCs) by standard methods at NCI. After selection with G418 and ganciclovir (Sigma), ESC clones were screened for correct homologous recombination by long-range PCR analysis (Fig. Supplement 1A-B). The PCR primers used to screen positive ESC clones are as follows: F1: 5-TACCCATCGTCTTCTTAAACCTCTT-3; F2: 5′-CAAACTGTCACGCCAAGATATC-3; F3: 5-CTCATGCGTTCTTGCTTCTTCACA-3; F4: 5-CGCATCGCCTTCTATCGCCTTCTT-3. The 5.7 kb PCR products from positive ES clones were digested with Sac I to confirm the correct knock-in of the upstream loxP site containing a Sac I recognition sequence (Fig. S1C). Mutant ESC clones were injected into C57/B6 carrier blastocysts and transplanted the same day into foster CD1 mothers. Chimeric offspring was bred with wild-type C57BL/6 mice (Charles River laboratories) to generate Lsh flox/+ mice. To delete the neomycin cassette flanked by Frt sites, the Lsh flox/+ mice were bred with actin-Flp transgenic mice (The Jackson Laboratory) and the pups were genotyped using the following primers: F5: 5-TTGTCCATAGAAAGATTGTGATTA-3; F6: 5-GAGTACCATCCACAGTTCTAAAAA-3. Lsh flox/+ mice were identified by PCR of the flanking region of the first loxP site using primer pairs: F7: 5-GTGCATTGGATCCTTTGTGGC-3 and F8: 5-CATTTCCACTTTAAAACACTCAG-3. Lsh flox/+ mice were maintained in a mixed genetic background from 129 Sv and C57Bl/6 J strains. To delete exon 9 and exon 10 Lsh flox/+ mice were

intercrossed with actin-Cre recombinase, Mx1-Cre recombinase or Vav-iCre recombinase transgenic mice (The Jackson Laboratory). Actin-Cre recombinase strain display Cre recombinase activity in all cells of the embryo by the blastocyst stage of development and delete the gene of interest in all tissues. The Mx1 strain has the Cre-recombinase under the control of the Mx1 promoter, which can be induced to high levels of transcription by administration of double-stranded RNA such as poly (I:C). Vav transgenic mice express Cre recombinase under the control of the mouse vav 1 promoter directing Cre recombinase expression to hematopoietic cells and their progenitors. CD45.1 C57BL/6 mice were purchased from Charles River laboratories. All mice were housed in the Frederick National Laboratory and treated with procedures approved by the National Institutes of Health Animal Care.

Poly (I:C) treatment and bone marrow transplantation

For non-competitive BMT (100% chimeras) mice from intercrossing with the Mx1 strain were grouped after genotyping and 10-week-old mice were intraperitoneally injected with 5 mg/kg of poly (I:C) (InvivoGen) every other day for three injections. Two weeks after the last injection, mice were euthanized, and bone-marrow cells were flushed from the tibias and femurs and washed three times in PBS. 8-week-old CD45.1+ recipients were irradiated with 10 Gy from a 137Cs source and reconstituted by lateral tail vein injection with $1x10^6$ freshly isolated bone marrow cells from donor mice.

For a competitive BMT (50:50 chimeras), we prepared bone marrow cells from Mx1 cre mice as described above. Briefly, 5×10^5 bone marrow cells carrying CD45.2 from 8-week-old Mx1 cre+ Lsh F/+(Ctrl) or Lsh F/F(KO) mice were mixed with equal amount of bone marrow cells from wild type congenic strain C57BL/6-Ly5.1 carrying CD45.1. These cells mixture was injected to lethally irradiated (10 Gy) female B6-Ly5.1 recipient mouse. Mice recovered 8 weeks prior to analysis.

B Cell Isolation and *in vitro* Cell Culture

B cells were isolated using immunomagnetic separation (Miltenyi Biotec) to sort for splenic $CD43^-$ $CD19^+$ $B220^+$ B cells. For enrichment of CD45.2+ B cells (donor) from spleens of CD45.1/CD45.2 (50 : 50 mixture) transplanted recipients, cell suspensions were first negatively isolated by depletion of CD45.1+ cell prior to B cell isolation. B cells were cultured in RPMI 1640

supplemented with 10% FBS, 10 μM β-mercaptoethanol, 1mM sodium pyruvate and 1× GlutaMAX(Thermo Fisher). Naive B cells were stimulated at 0.5 × 10^6/mL density to induce CSR to IgG3 (25 µg/mL LPS) or IgG1 (25 µg/mL LPS and 25 ng/mL recombinant IL-4) or IgG2a(25 µg/mL LPS and 20 ng/mL IFNγ), CSR efficiency was analyzed by FACS after 3 days of stimulation.

EdU Cell Proliferation and CFSE Dilution Assay

Purified B cells were seeded into 6-well plates with a cell density of 1 million per well. cells were stimulated with LPS and IL4 for 3 days and counted again. 1 million cells were harvested and labeled with 10μM EdU in medium for 4 hours in 37C° incubator. Cells were spin down at 300g for 5 min and fixed in Thermo Fisher Fix/perm solution (00-5123-43) for 15 min at room temperature. Then the cells were incubated with eBioscience Permeabilization Buffer (00-8333-56) for another 15 min and was washed by cold PBS once. Azide-Alex488 fluorescent dye click it reaction buffer was prepared according to manufacture（C10276). Cells was incubated with this reaction buffer for 30 min at room temperature and was washed by 1 ml cold PBS once. Then the cells were subjected to flow cytometry analysis.

For the CFSE dilution assay, purified spleen B cells were pulsed with 5 µM CFSE (Life Technologies), washed, and cultured in the presence of LPS plus IL- 4. Cells were collected 4 days after CFSE pulsing and IgG1 CSR stimulation, stained with anti-mouse IgG1 APC conjugation. The concentration of pulsed CFSE decreases as the cells divide, and thus the fluorescent intensity of CFSE reflects the stages of cell division.

Cell Apoptosis assay

Harvest the cells and resuspend 5× 10^5 cells in 200 µL of Annexin V binding buffer containing 5 ul of Annexin V-FITC and incubate for 10 min at room temperature. Then cells were washed in 200 µL Binding Buffer (1x) and resuspend in 190 µL binding buffer (1x). 10 µL Propidium Iodide (20 µg/mL) was added to the cell suspension. Samples were ready for FACS analysis.

Fluorescent Inhibitor Based Active Poly Caspases Detection Assay

Cells were harvested and resuspended to a concentration of 1×10^6 cells/mL in culture media. 300 µL of cell suspension was transferred to flow tubes, 10 µL of 30X FLICA(V35117) working solution

was directly added to the 300 µL cell suspension. Cells were mixed by flicking tubes and then was incubated 60 minutes at 37C° and 5% CO2, protected from light. Make sure to mix the tubes twice during incubation to minimize cell settling. Applying 2ml 1X wash buffer to each tube to wash samples. Pellet the cells by centrifugation. Cells were resuspended in 1 mL of wash buffer and wash again. The supernatant was discarded, and cells were resuspended in 400 µL of 1X wash buffer. 2 µL of propidium iodide was added to the cell suspension and incubate for 10 minutes on ice. The samples were ready for FACS analysis.

DC-PCR analysis

The DC–PCR assay was performed as previously described (1). In brief, genomic DNA was isolated from day 4 LPS plus IL4 stimulated B cells and subsequently purified using Blood and tissue kit (QIAGEN). Five micrograms of genomic DNA were digested overnight with 20 U of EcoRI (New England biolab). Ligations were performed under diluted conditions to promote circularization. Digested DNA was ligated overnight at 16°C with a concentration of 1.8 ng/µl in a total volume of 100 µl per reaction. Three to four ligation reactions were pooled, ultrafiltration concentrated and serially diluted at a 1:2 ratio prior PCR analysis. PCR analysis was performed in 50 µl per reaction using standard protocol of MyTaq polymerase (Bioline) with serially diluted DNA. Primers were designed to amplify the Sµ-Sγ1 rearrangements that occur during CSR to IgG1 in direct chromosomal joining of Sµ-Sγ1 with excision of circular. As a control for EcoRI digestion and circularization of input DNA, amplification of an EcoRI fragment of nicotinic acetylcholine receptor B subunit gene (nAChR) was performed, which, after EcoRI digestion and circularization, generates a 753-bp DC–PCR product. Primers are listed in Supplemental Table 1.

Biotin-Labeling DNA Break Assay.

The biotin-labeling DNA break assay was performed as described previously (2) with a slight modification. To remove dead cells, 5 million cells were layered on lymphocytes separation medium (Lonza, 1.077g/mL) and centrifuged at 1,500 g for 25 min at room temperature. Live cells were collected from the interface between the two layers. Cells were washed in cold PBS and then fixed with 1 mL of fixation buffer (1.5g of bronopol, 1.5g of diazolidinyl urea, 0.6 g of zinc sulfate heptahydrate, 0.145 g of sodium citrate dihydrate and 50 mM EDTA in 50 mL H2O) for 15

min at room temperature. Then the cells were washed again in cold PBS and sequentially resuspended into cold buffer A [0.25% TritonX-100, 10 mM EDTA, 10 mM Hepes (pH 6.5)], cold buffer B [200 mM NaCl, 1 mM EDTA, 10 mM Hepes (pH 6.5)]. Nuclei were permeabilized with buffer C [100 mM Tris-HCl (pH 7.4), 50 mM EDTA, 1% TritonX-100] for 30 min on ice. Cells were sequentially washed in cold PBS and TdT buffer, then resuspended into 100 μL of TdT buffer with 3 μL of 1 mM biotin-16-dUTP (Biotium) and 60 U of TdT (New England Biolabs), then incubated for 1 h at 37 °C. After that, the cells were washed with buffer D [100 mM Tris-HCl (pH 7.4), 150 mM NaCl], resuspended into lysis buffer [10 mM EDTA, 10 mM Tris-HCl (pH 8.0), 150 mM NaCl, 0.1% SDS, 0.2 mg/mL of proteinase K], and then incubated overnight at 56 °C. Genomic DNA was isolated by phenol/chloroform extraction. Genomic DNA (10mg) was digested with HindIII overnight at 37 °C. Biotinylated fragments were captured with 20 μL of streptavidin magnetic beads (Dynabeads™ MyOne™ Streptavidin C1). The particles were washed with 300 μL of TE 3 times and then resuspended into 30 μL of 10 mM Tris-HCl (pH 8.0). Two μL of the beads was used for PCR, and 1μL of Hind III digested DNA for each sample was used as input. PCR reactions were set up following standard protocol of Q5 hot start polymerase from New England Biolabs. Primer sequences are provided in table 1.

END-seq analysis

End seq analysis was performed as previously described (3). B Cells were stimulated with LPS, IL4 and anti-CD180 for 48 hours. About 15 million B cells in single cell suspension were embedded in a single agarose plug. Agarose plugs were treated with Proteinase K and RNase A to recover DNA. After blunting and A-tailing the DNA ends, DNA was ligated to a biotinylated hairpin adaptor containing a 3′ T overhang and Illumina's p5 sequence. Subsequently, the agarose plugs were melted, and DNA was extracted and sheared, and biotinylated DNA enriched over streptavidin-coated beads. The new ends created by sonication were also end repaired and A-tailed, allowing ligation of a second hairpin adaptor containing Illumina's p7 sequence which was used for subsequent library amplification and high throughput sequencing. The number of mapped reads is thought to correlate with the frequency of DNA ends in the cell population. Supplemental Table S2 displays normalized tag numbers for the immunoglobulin heavy chain region for CTRL and KO samples presented as sum or mean per 5kb bins across the genome (MM10 annotation).

RT-PCR analysis

mRNA levels for AID, post-spliced germline transcripts, and post-switch transcripts were measured as previously described (4). Total RNA was extracted from purified CD19+ B cells stimulated 24h for detection of germline transcripts or 72h for detection of AID and post-transcripts using RNeasy Mini Kit (QIAGEN) and reverse-transcribed with iScript cDNA Synthesis Kit (Biorad). Transcripts were amplified using MyiQ2 two colors Real-Time PCR detection system (Biorad) with iTaq Universal qPCR Mastermix (Biorad). Primers used for qPCR are listed in Supplemental Table 1.

Linear Amplification-Mediated High-throughput genome-wide translocation sequencing (LAM-HTGTS)

LAM-HTGTS libraries were generated as previously described (5). Briefly, genomic DNA was extracted from cultured mouse splenic B cell and subjected to sonication to generate DNA fragment with average size of 750bp. Then LAM-PCR was performed using a single biotinylated primer (CAGACCTGGGAATGTATGGT) for 80 cycles using Phusion polymerase (thermo scientific). Biotinylated DNA fragments were captured with Dynabeads MyOne streptavidin C1 beads (Invitrogen) at room temperature for 4 hr, followed by on-bead ligation at 25°C with bridge adapters for more than 6 hr in the presence of 15% PEG-8000 (Sigma) and 1 mM hexammine cobalt chloride (Sigma). After washing beads with 1X B&W buffer three times, ligated products were subjected to 17 cycles of on-beads PCR with Phusion polymerase followed by PCR purification Kit (Qiagen). In order to add Illumina Miseq-compatible adapters at 5’ and 3’ ends, a third round of tagging-PCR was carried out for another 16 cycles with Phusion polymerase. PCR products were size separation for DNA fragments between 500-1000 bp on the 1% agarose gel and apply to column purification using QIAquick gel extraction Kit (Qiagen) before loading onto Illumina Miseq machine for sequencing. Samples were processed through HISAT (6) to align to reference mouse genome MM10 and duplicates were excluded from analysis. Output was for Sequence/Binary Alignment Map (SAM/BAM) (7) files and parsed and analyzed using custom programs. CTRL samples and KO samples were pooled and samples with suggested insertions of nucleotides were excluded from analysis. Samples with microhomology $\geqslant 1$ were further

processed with the UCSC genome browser BLAT program to determine junctions. Samples were also processed through Lumpy (8) software to detect structural variants by using the lumpyexpress program. Lumpy produces output in variant call format (VCF) files and SAM files and suggested breakpoints are depicted in Fig5E.

ChIP-qPCR assay

ChIP (chromatin immunoprecipitation) assays were carried out as described (9). Briefly, cells were cross-linked with 1% formaldehyde, lysed, and sonicated on ice to generate DNA fragments with an average length of 200-800 bp. 1% of each sample was saved as input fraction. Immunoprecipitation was performed using gamma-H2AX antibody (Abcam, ab2893) or IgG (Millipore, 12-370) as control. After reversal of cross-linking, precipitated DNA was suspended in 50 µl of Nuclease-Free water and analyzed by qPCR using the specific primers shown in Supplemental Table 1. The normalization method for ChIP analysis is percent of input. Each ChIP result represents the average of four samples (mean ± SD).

Flow cytometry and cell sorting

We analyzed single-cell suspensions from various mice organs by flow cytometry using antibodies (eBioscience) conjugated with PE, PE-Cy5, PE-Cy7, FITC, APC, APCeFlour780, eFluor450 or Pacific Blue: Mac-1/CD11b (17-0112-82), Gr-1 (12-5931-82), CD8 (17-0081-82), CD4 (12-0042-85), CD71 (17-0711-82), TER119 (12-5921-82), B220 (12-0452-83), CD19 (17-0193-82), IgM (12-5890-82), Mouse Hematopoietic Lineage eFluor450 Cocktail kit (88-7772-72), Sca1 (17-5981-82), c-Kit (12-1172-82), Flt3 (15-1351-81), CD45.2 (11-0454-82). For EdU incorporation and apoptosis assay, EdU Staining Kit (eBioscience) and Annexin-V/PI and 7-AAD staining kit (Thermo Fisher) were used according to the manufacturer's instructions. We analyzed all samples on a FACSCanto, LSR II cytometer (BD Biosciences), or sorted them on a FACSAria (BD Biosciences) using standard protocols and analyzed the data with FlowJo software.

Enzyme-linked immunosorbent assay

Mouse serum IL-4, IFN-γ, IL-2, IL-6, IL10 and IL-1β cytokines were measured using ELISA kits (R&D and eBioscience) following the manufacturer's instructions. Mouse serum or IL-4/LPS stimulated

splenocytes supernatants Ig isotypes were determined using Mouse Ig Isotyping ELISA Kit (eBioscience, 88-50630-86) according to the manufacturer's instructions.

GFP based chromosomal DSB NHEJ repair assay

For the DSB repair assays (10), U2OS cells (a kind gift from Dr Jeremy Stark) were seeded at a cell density of 0.2 × 10^5 cells per well of a 12-well plate. Each well was firstly transfected with 3.75 pmol of siRNA together 3 µl of RNAiMAX for 2 days, then transfected with 1ug of each sgRNA/Cas9 plasmid (a kind gift from Dr Jeremy Stark) using 4 µl of PEI in 1ml complete culture medium. Cells were analyzed by flow cytometry days post transfection. The frequency of GFP+ cells was normalized to transfection efficiency, which was measured using parallel transfections with a DsRed-expression vector (Clonetech). For example, if a Cas9/sgRNA transfection resulted in 3% GFP+ cells, and the transfection frequency for that cell line in the parallel experiment is 30% DsRed+, then the normalized repair frequency is 10% GFP+. We also used Cas9-PE antibody (CST) to stain intracellular Cas9 protein to assess cells successfully transfected. In this way, GFP+ cells are normalized to Cas9-PE+ cells. Each bar represents the mean of at least 3 independent transfections, error bars represent standard deviation, and statistics are as described in the figure legends.

Statistical analysis

Statistical analyses were performed with GraphPad Prism software by using unpaired, 2-tailed Mann-Whitney test unless otherwise stated. Data represent the mean ± SD, $P < 0.05$ was considered a statistically significant difference.

References

1. Dong J, et al. (2015) Orientation-specific joining of AID-initiated DNA breaks promotes antibody class switching. Nature 525(7567):134-139.

2. Doi T, et al. (2009) The C-terminal region of activation-induced cytidine deaminase is responsible for a recombination function other than DNA cleavage in class switch recombination. Proceedings of the National Academy of Sciences of the United States of America 106(8):2758-2763.

2. Doi T, et al. (2009) The C-terminal region of activation-induced cytidine deaminase is responsible for a recombination function other than DNA cleavage in class switch recombination. Proceedings of the National Academy of Sciences of the United States of America 106(8):2758-2763.

4. Muramatsu M, et al. (2000) Class switch recombination and hypermutation require activation-induced cytidine deaminase (AID), a potential RNA editing enzyme. Cell 102(5):553-563.

5. Hu J, et al. (2016) Detecting DNA double-stranded breaks in mammalian genomes by linear amplification-mediated high-throughput genome-wide translocation sequencing. Nature protocols 11(5):853-871.

6. Kim D, Langmead B, & Salzberg SL (2015) HISAT: a fast spliced aligner with low memory requirements. Nature methods 12(4):357-360.

7. Li H, et al. (2009) The Sequence Alignment/Map format and SAMtools. Bioinformatics (Oxford, England) 25(16):2078-2079.

8. Layer RM, Chiang C, Quinlan AR, & Hall IM (2014) LUMPY: a probabilistic framework for structural variant discovery. Genome biology 15(6):R84.

9. Nelson JD, Denisenko O, & Bomsztyk K (2006) Protocol for the fast chromatin immunoprecipitation (ChIP) method. Nature protocols 1(1):179-185.

10. Bhargava R, et al. (2018) C-NHEJ without indels is robust and requires synergistic function of distinct XLF domains. Nat Commun 9(1):2484.

2. Table S1. Primer design

Primer	5'-3'
**germline transcript**	
Ig1_F	GGCCCTTCCAGATCTTTGAG
Cg1_R	GGATCCAGAGTTCCAGGTCACT
Ig3_F	TGGGCAAGTGGATCTGAACA
Cg3_R	CTCAGGGAAGTAGCCTTTGACA
Ig2a_F	GGCTGTTAGAAGCACAGTGACAAAG
Cg2a_R	CACATTGCAGGTGATGGtcTGG
**post-switch transcript**	
Ighj4_F	ATTACTATGCTATGGACTACTGGGG
Cg1_R	GGATCCAGAGTTCCAGGTCACT
Cg3_R	CTCAGGGAAGTAGCCTTTGACA
Cg2a_R	CACATTGCAGGTGATGGtcTGG
Ce_R	CCAGGGTCATGGAAGCAGTG
AID_F	AGATAGTGCCACCTCCTGCTCACTGG
AID_R	GGCTGAGGTTAGGGTTCCATCTCAG
**ChIP**	
Sμ_F	CAATGTGGTTTAATGAATTTGAAGTTGCCA
Sμ_R	TCTCACACTCACCTTGGATCTAAGCACTGT
Sγ1_F	AGTGTGGGAACCCAGTCAAA
Sγ1_R	GTACTCTCACCGGGATCAGC
**DC-PCR**	
DC_Sγ1_F	CATGAGAGCTGGAGCTAGTATGAAGGTG
DC_Sγ1_R	ACTGACTGACTGAGTGTCCTCTCAAC
nAChR_F	GACTGCTGTGGGTTTCACCCAG
nAChR_R	GGCGCGCACTGACACCACTAAG

3. Table S2. Read numbers of CTRL and KO samples derived by End-seq analysis for the IgH constant region locus (MM10)

chr	start	end	ctrl sum	ctrl mean	KO sum	KO mean
chr12	113130000	113134999	93.4248	0.0187	43.5001	0.0087
chr12	113135000	113139999	56.9602	0.0114	11.7818	0.0024
chr12	113140000	113144999	83.8686	0.0168	39.7140	0.0079
chr12	113145000	113149999	148.5304	0.0297	79.4280	0.0159
chr12	113150000	113154999	49.5101	0.0099	103.2564	0.0207
chr12	113155000	113159999	172.3895	0.0345	275.6946	0.0551
chr12	113160000	113164999	145.8898	0.0292	81.4137	0.0163
chr12	113165000	113169999	176.7590	0.0354	75.2448	0.0150
chr12	113170000	113174999	96.1597	0.0192	164.9720	0.0330
chr12	113175000	113179999	133.5044	0.0267	86.7619	0.0174
chr12	113180000	113184999	174.4643	0.0349	131.0032	0.0262
chr12	113185000	113189999	191.0305	0.0382	83.3994	0.0167
chr12	113190000	113194999	115.1464	0.0230	68.2551	0.0137
chr12	113195000	113199999	123.6339	0.0247	69.4465	0.0139
chr12	113200000	113204999	167.2028	0.0334	53.6139	0.0107
chr12	113205000	113209999	138.8798	0.0278	184.6701	0.0369
chr12	113210000	113214999	195.6829	0.0391	190.7861	0.0382
chr12	113215000	113219999	216.6500	0.0433	151.4692	0.0303
chr12	113220000	113224999	193.1995	0.0386	138.4430	0.0277
chr12	113225000	113229999	96.4740	0.0193	142.2291	0.0284
chr12	113230000	113234999	162.6761	0.0325	131.8505	0.0264
chr12	113235000	113239999	35.3644	0.0071	116.4414	0.0233
chr12	113240000	113244999	75.4440	0.0151	99.1526	0.0198
chr12	113245000	113249999	113.1660	0.0226	99.1526	0.0198
chr12	113250000	113254999	94.3050	0.0189	110.0872	0.0220
chr12	113255000	113259999	79.5934	0.0159	128.8587	0.0258
chr12	113260000	113264999	49.5101	0.0099	75.4566	0.0151
chr12	113265000	113269999	136.0821	0.0272	101.2707	0.0203
chr12	113270000	113274999	123.1309	0.0246	75.4566	0.0151
chr12	113275000	113279999	63.4987	0.0127	95.3136	0.0191
chr12	113280000	113284999	150.8880	0.0302	113.0260	0.0226
chr12	113285000	113289999	96.6626	0.0193	105.4010	0.0211
chr12	113290000	113294999	44.7006	0.0089	73.5239	0.0147
chr12	113295000	113299999	148.9705	0.0298	133.9686	0.0268
chr12	113300000	113304999	172.8925	0.0346	113.1849	0.0226
chr12	113305000	113309999	172.1066	0.0344	77.4423	0.0155
chr12	113310000	113314999	124.7341	0.0249	152.7930	0.0306
chr12	113315000	113319999	145.9213	0.0292	162.5362	0.0325
chr12	113320000	113324999	188.4528	0.0377	162.6685	0.0325
chr12	113325000	113329999	300.4557	0.0601	262.0859	0.0524
chr12	113330000	113334999	814.0093	0.1628	1573.8660	0.3148

chr12	113335000	113339999	1654.8329	0.3310	2762.5056	0.5525
chr12	113340000	113344999	302.4990	0.0605	396.3722	0.0793
chr12	113345000	113349999	198.8578	0.0398	190.3360	0.0381
chr12	113350000	113354999	115.5236	0.0231	137.6222	0.0275
chr12	113355000	113359999	89.5898	0.0179	77.4423	0.0155
chr12	113360000	113364999	67.4281	0.0135	297.8550	0.0596
chr12	113365000	113369999	148.4046	0.0297	125.0991	0.0250
chr12	113370000	113374999	188.6729	0.0377	178.4747	0.0357
chr12	113375000	113379999	262.6080	0.0525	184.9084	0.0370
chr12	113380000	113384999	147.9960	0.0296	210.4842	0.0421
chr12	113385000	113389999	188.8929	0.0378	168.3874	0.0337
chr12	113390000	113394999	133.4730	0.0267	203.4945	0.0407
chr12	113395000	113399999	94.2107	0.0188	97.8553	0.0196
chr12	113400000	113404999	251.9515	0.0504	104.6596	0.0209
chr12	113405000	113409999	200.3981	0.0401	151.6810	0.0303
chr12	113410000	113414999	240.4778	0.0481	137.0133	0.0274
chr12	113415000	113419999	322.3345	0.0645	436.4039	0.0873
chr12	113420000	113424999	900.1727	0.1800	1451.7850	0.2904
chr12	113425000	113429999	1784.6606	0.3569	2951.2005	0.5902
chr12	113430000	113434999	21.1243	0.0042	0.0000	0.0000
chr12	113435000	113439999	23.5763	0.0047	47.6303	0.0095
chr12	113440000	113444999	58.7835	0.0118	65.3957	0.0131
chr12	113445000	113449999	21.2186	0.0042	3.9714	0.0008
chr12	113450000	113454999	25.9339	0.0052	43.6854	0.0087
chr12	113455000	113459999	2.3576	0.0005	15.8856	0.0032
chr12	113460000	113464999	9.4305	0.0019	57.0028	0.0114
chr12	113465000	113469999	16.5034	0.0033	51.6282	0.0103
chr12	113470000	113474999	40.0796	0.0080	40.2435	0.0080
chr12	113475000	113479999	56.4573	0.0113	40.7995	0.0082
chr12	113480000	113484999	7.0729	0.0014	55.7585	0.0112
chr12	113485000	113489999	62.9957	0.0126	61.5567	0.0123
chr12	113490000	113494999	89.4954	0.0179	140.0580	0.0280
chr12	113495000	113499999	89.3697	0.0179	59.4651	0.0119
chr12	113500000	113504999	181.1599	0.0362	75.4566	0.0151
chr12	113505000	113509999	46.9639	0.0094	125.0197	0.0250
chr12	113510000	113514999	66.0135	0.0132	135.2129	0.0270
chr12	113515000	113519999	80.1593	0.0160	17.8713	0.0036
chr12	113520000	113524999	21.2186	0.0042	75.4566	0.0151
chr12	113525000	113529999	71.9547	0.0144	45.6711	0.0091
chr12	113530000	113534999	100.2148	0.0200	59.3857	0.0119
chr12	113535000	113539999	61.2354	0.0122	59.4916	0.0119
chr12	113540000	113544999	70.1629	0.0140	29.7855	0.0060
chr12	113545000	113549999	61.3297	0.0123	47.6568	0.0095
chr12	113550000	113554999	77.4558	0.0155	90.9715	0.0182

4. Supplemental Figure Legends

Fig S1. Generation of Lsh conditional knockout mice. A. Murine Lsh locus on Chr 19 with the targeted locus and primer location for screening. B-D. Genomic DNA long-range PCR analysis to determine the correct homologous recombination at downstream region using primer pair F1/F2 (B) and upstream sequence using primer pair F3/F4 (C) in ES cell clones. PC, Lsh targeted positive clone; NC, non-targeted negative clone; M, DNA marker. The 5.7 kb PCR amplification product derived from positive ES clones was gel purified and digested with Sac I to confirm the correct position of the upstream loxP site (D). +, ES clone with correct upstream loxP site; -, ES clone without upstream loxp site. E. Western analysis of embryonic brain tissue to validate the deletion of Lsh in Lsh KO mice after crossbreeding of floxed Lsh Cko mice with actin-Cre-recombinase transgenic mice. F. Mating of heterozygous mice harboring a wild type and a conditional knockout allele with a deletion of exon 9 and 10.

Fig S2. Characterization of hematopoietic stem cells after Lsh depletion in bone marrow. A-B. Flow cytometry analysis of Lin- Sca-1+ c-Kit+ (LSK+) cells in the bone marrow from Lsh (F/F) Mx1-cre- (Ctrl) and Lsh (F/F) Mx1-cre+ (KO) after two weeks of poly (I:C) treatment. B. Summary of LSK+ cells in BM of Mx1-cre- (Ctrl) and Lsh (F/F) Mx1-cre+ (KO) mice. C. Schematic graph showing the bone marrow transplantation (BMT) protocol. Two weeks after poly (I:C) injection of the Ctrl and KO mice, BMT was performed using lethally irradiated wild-type recipient mice . Quantitative data was presented as mean$\pm$SD and were analyzed using two-tailed unpaired Mann-Whitney test. ns, not significant.

Fig S3. Hematopoietic development in peripheral blood and lymphoid organs after non-competitive BMT (100% chimera) in recipient mice. Cre-recombinase was induced by injection of poly (I:C) to generate Lsh KO mice (Mx1$^{Cre/+}$Lsh $^{fl/fl}$ with poly (I:C) administered) and control mice (Mx1$^{Cre/+}$Lsh $^{fl/+}$ with poly (I:C) administered) and non-competitive BMT (100% donor cells) was conducted. FACS analysis was performed for detection of CD45.2^{+} donor cells in irradiated CD45.1^{+} recipient mice one (1M), two(2M) or three (3M) months after BMT. A-B. FACS analysis for detection of Mac1^{+}GR1^{+} cells among CD45.2^{+} cells in peripheral blood (PB). C-E. FACS analysis for detection of CD4^{+} and CD8^{+} cells among CD45.2^{+} cells in peripheral blood (PB). F-I. FACS analysis for detection of CD4^{+} and CD8^{+} T cell subsets in the spleen. Relative frequency among

CD45.2$^+$ donor cells (F,H) and absolute numbers for each subset (G,I). J. Total number of thymocytes. K. Proportion of CD45.2$^+$ donor cells in the thymus. L.-Q. FACS analysis for detection of CD4$^+$ / CD8$^+$ T cell subsets among CD45.2$^+$ cells in thymus. Relative frequency among CD45.2$^+$ donor cells (L, N, P) and absolute numbers for each subset (M, O, Q). Control cells (●) or Lsh KO cells (■). n=5-8 per group for FACS analysis. Quantitative data was presented as mean$\pm$SD and were analyzed using two-tailed unpaired Mann-Whitney test. ns, not significant, *$p < 0.05$, **$p < 0.01$ and *** $p < 0.001$.

Fig S4. Hematopoietic development in peripheral blood and lymphoid organs after competitive BMT (50:50 chimera) in recipient mice. Cre-recombinase was induced by injection of poly (I:C) to generate Lsh KO mice (Mx1$^{Cre/+}$Lsh $^{fl/fl}$ with poly (I:C) administered) and control mice (Mx1$^{Cre/+}$Lsh $^{fl/+}$ with poly (I:C) administered) and competitive BMT (50% donor cells from Lsh KO or control mice were mixed with 50% wild type cells) was conducted. FACS analysis was performed for detection of CD45.2$^+$ donor cells in irradiated CD45.1$^+$ recipient mice at two months after BMT. A. FACS analysis for detection of CD45.2$^+$ cells in peripheral blood. B.-E. FACS analysis for detection of CD19$^+$B220$^+$ (B), Mac1$^+$GR1$^+$ cells (C), CD8$^+$ (D) and CD4$^+$ (E) cells among CD45.2$^+$ cells in peripheral blood (PB). F. FACS analysis for detection of CD45.2$^+$ cells in spleen. G.-L. FACS analysis for detection of relative frequency and absolute numbers of CD19$^+$B220$^+$ (G,H) and CD4$^+$ (I,J) and CD8$^+$ (K,L) cells among CD45.2$^+$ cells in the spleen. M. Total cell population in BM. N. FACS analysis for detection of CD45.2$^+$ cells in BM. O.-P. FACS analysis for detection of relative frequency (O) and total population (P) of CD19$^+$B220$^+$ in BM. Q. Total cell population in thymus. R. FACS analysis for detection of CD45.2$^+$ cells in thymus. S.-U. FACS analysis for detection of indicated T cell subsets in thymus. Control cells (●) or Lsh KO cells (■). n=6-10 per group for FACS analysis. Quantitative data was presented as mean$\pm$SD and were analyzed using two-tailed unpaired Mann-Whitney test. ns, not significant, *$p < 0.05$, **$p < 0.01$ and *** $p < 0.001$.

Fig S5. Growth and apoptosis assessment of purified Lsh deficient B cells after BMT. A. Schematic graph showing the bone marrow transplantation (BMT) protocol for competitive BMT (50:50 chimeras). Two weeks after poly (I:C) injection of the control (Ctrl) and Mx1 KO (KO) mice, BMT was performed using lethally irradiated wild-type recipient mice. After two months B cells were purified from spleen and cultured for three days with LPS and IL-4. B. C. FACS

analysis for detection of EdU incorporation. D. FACS analysis measuring 7-AAD to determine cell death. E. F. FACS analysis to determine the proportion of Propidium iodide (PI) and caspase (Cas) positive cells. N=4 animals were used for each group. Quantitative data was presented as mean$\pm$SD and were analyzed using two-tailed unpaired Mann-Whitney test. ns, not significant, *$p < 0.05$.

Fig S6. Determination of immunoglobulin secretion in the absence of Lsh. A-F. ELISA analysis of indicated immunoglobulin isotypes one months (1M), two months (2M) or three months (3M) after BMT (100% chimera) of Lsh F/F Mx1-cre+ (KO) and Lsh F/+ Mx1-cre+ control mice (Ctrl). Quantitative data was presented as mean$\pm$SD and were analyzed using two-tailed unpaired Mann-Whitney test. ns, not significant, *$p < 0.05$, **$p < 0.01$ and *** $p < 0.001$.

Fig S7. Serum immunoglobulin deficiency in mice with Lsh depletion in HSPCs and the short-term effect of Lsh deletion on hematopoiesis. A.-B. ELISA analysis of indicated Ig isotypes in the serum of Lsh F/F Mx1-cre+ (KO) and Lsh F/+ Mx1-cre+ (Ctrl) mice at age of 12 weeks (A) and 20 weeks (B) without BMT, n=8 per group. C-F. FACS analysis for detection of indicated immune cells in the bone marrow (C), spleen (D), thymus (E) and Peripheral blood (F) from Lsh F/F Mx1-cre+ (KO) and Lsh F/+ Mx1-cre+ (Ctrl) mice after two weeks of poly (I:C) injection, n=4-5 per group. Quantitative data was presented as mean$\pm$SD and were analyzed using two-tailed unpaired Mann-Whitney test. ns, not significant, *$p < 0.05$ and *** $p < 0.001$.

Fig S8. Normal cytokine production in mice with Lsh depletion in HSPCs. ELISA analysis for detection of IL-2 (A), IL-4 (B), IL-1β (C), IFNγ (D), IL-6 (E), IL-10 (F) in the serum of Lsh F/F Mx1-cre+ (KO) and Lsh F/+ Mx1-cre+ (Ctrl) mice 2 weeks (pre-BMT) or 10 weeks (pre-BMT2) after poly (I:C) injection or after one month (BMT-1M) or three months (BMT-3M) of BMT, n=4-8 per group. Quantitative data was presented as mean$\pm$SD and were analyzed using two-tailed unpaired Mann-Whitney test.

Fig S9. Growth and apoptosis assessment of purified Lsh deficient B cells without BMT. Purified B cells derived from Lsh F/F Vav-cre+ (KO) and Lsh F/+ Vav-cre+ (Ctrl) mice were cultured for three days supplemented with LPS and IL-4. A. Total cell numbers. n=9 for each group. B.C. FACS for detection of EdU incorporation. n=4 for each group D. FACS analysis for detection of early

apoptosis and cell death assessing Annexin V/ Propidium iodide (PI) double staining. n=8 for each group. E. FACS analysis for detection of late apoptosis and cell death assessing caspase activation (Cas)/ Propidium iodide (PI) double staining. n=8 for each group. Quantitative data was presented as mean$\pm$SD and were analyzed using two-tailed unpaired Mann-Whitney test. ns, not significant.

Fig S10. End-Seq analysis and DC-PCR analysis. A. Frequency of DSBs detected by End-seq analysis two days after stimulation with LPS+IL4 of purified $CD19^+$ B cells derived from Lsh F/F Vav-cre+ (KO) and Lsh F/+ Vav-cre+ (Ctrl) mice. B. DC–PCR results from two additional independently derived samples from LPS and IL-4-activated CD19+B cells of Lsh F/F Vav-cre+ (KO) and Lsh F/+ Vav-cre+ (Ctrl) mice as depicted in Figure 5B. Quantitative data was presented as mean$\pm$SD and were analyzed using two-tailed unpaired Mann-Whitney test. *$p < 0.05$.

Fig S11. End joining assay. A-C. USO2 GFP reporter cells were first treated with siLsh or siCtrl for 48 hrs and then transfected with expression plasmids for Cas9 and guiding RNAs or a control plasmid (DsRed) and analyzed by flow cytometry after 72 hrs. D. USO2 GFP reporter cells were first treated with siLsh or siCtrl for 48 hrs and then transfected with expression plasmids for Cas9 and guiding RNAs. Cells were stained for Cas9 and analyzed by flow cytometry after 72 hrs. Summary of n=9 biologic replicates for each group. Quantitative data was presented as mean$\pm$SD and were analyzed using two-tailed unpaired Mann-Whitney test. *** $p < 0.001$.

5. Supplemental Figures

SI Fig S1

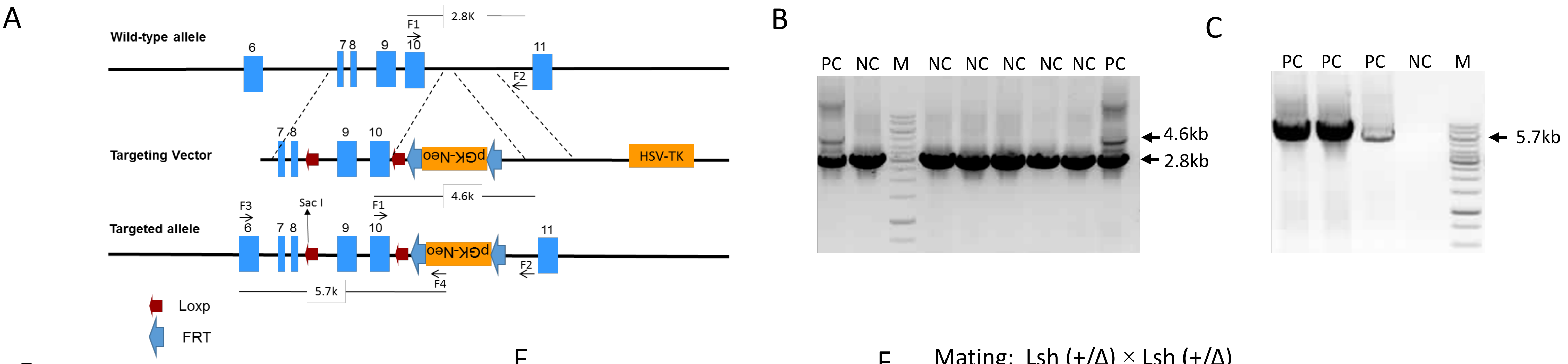

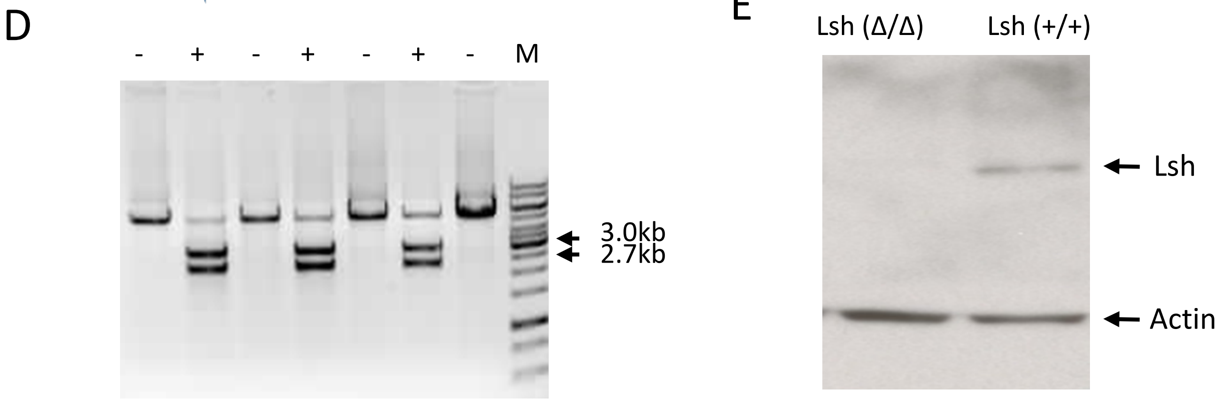

F

Mating: Lsh (+/Δ) × Lsh (+/Δ)

Genotype	# Alive pups
Lsh (+/+)	35
Lsh (+/Δ)	61
Lsh (Δ/Δ)	0

SI Fig S2

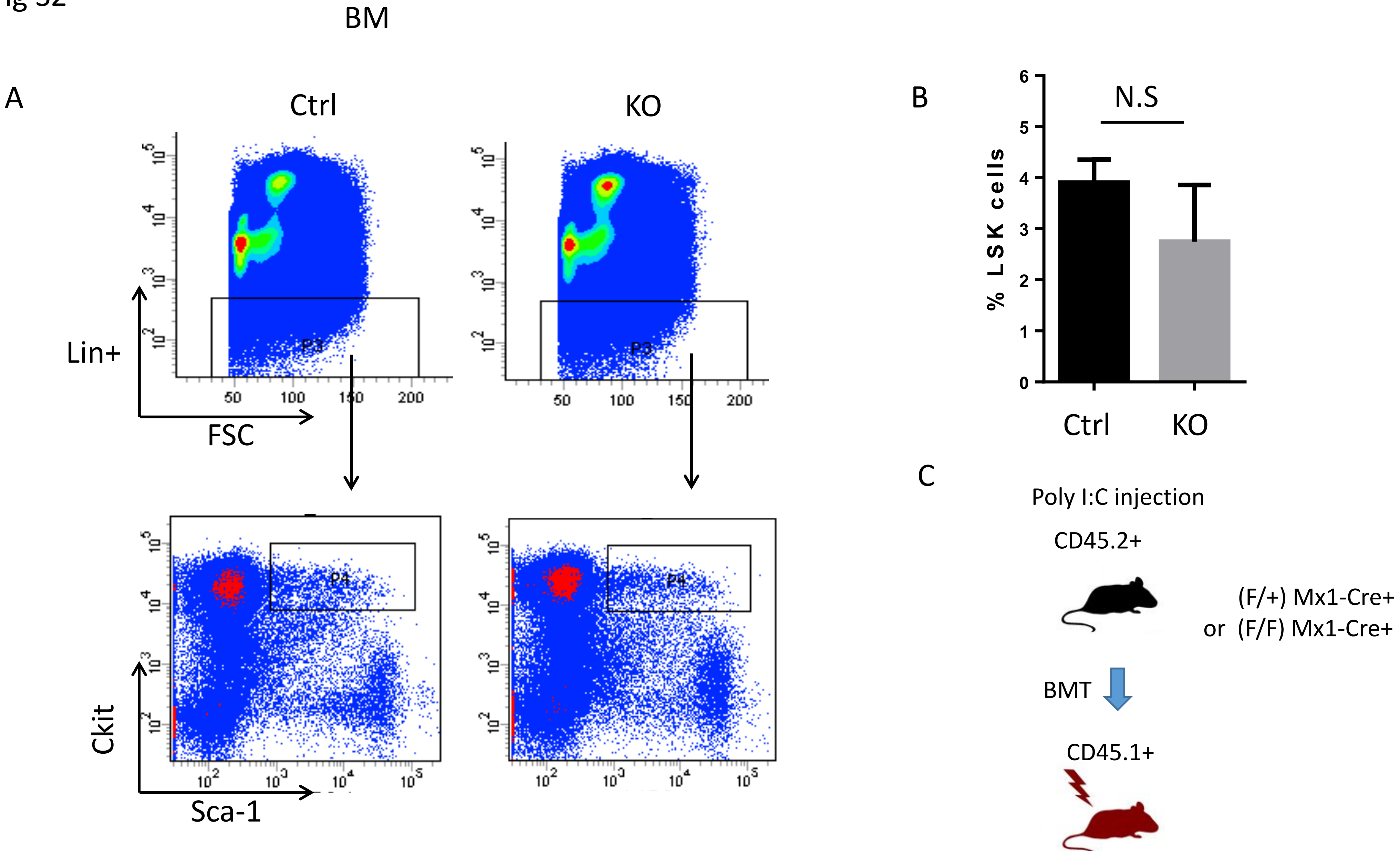
BM
A
Ctrl
KO
Lin+
FSC
P3
Ckit
Sca-1
P4
B
N.S
% LSK cells
0
1
2
3
4
5
6
Ctrl
KO
C
Poly I:C injection
CD45.2+
(F/+) Mx1-Cre+
or (F/F) Mx1-Cre+
BMT
CD45.1+

SI Fig S3

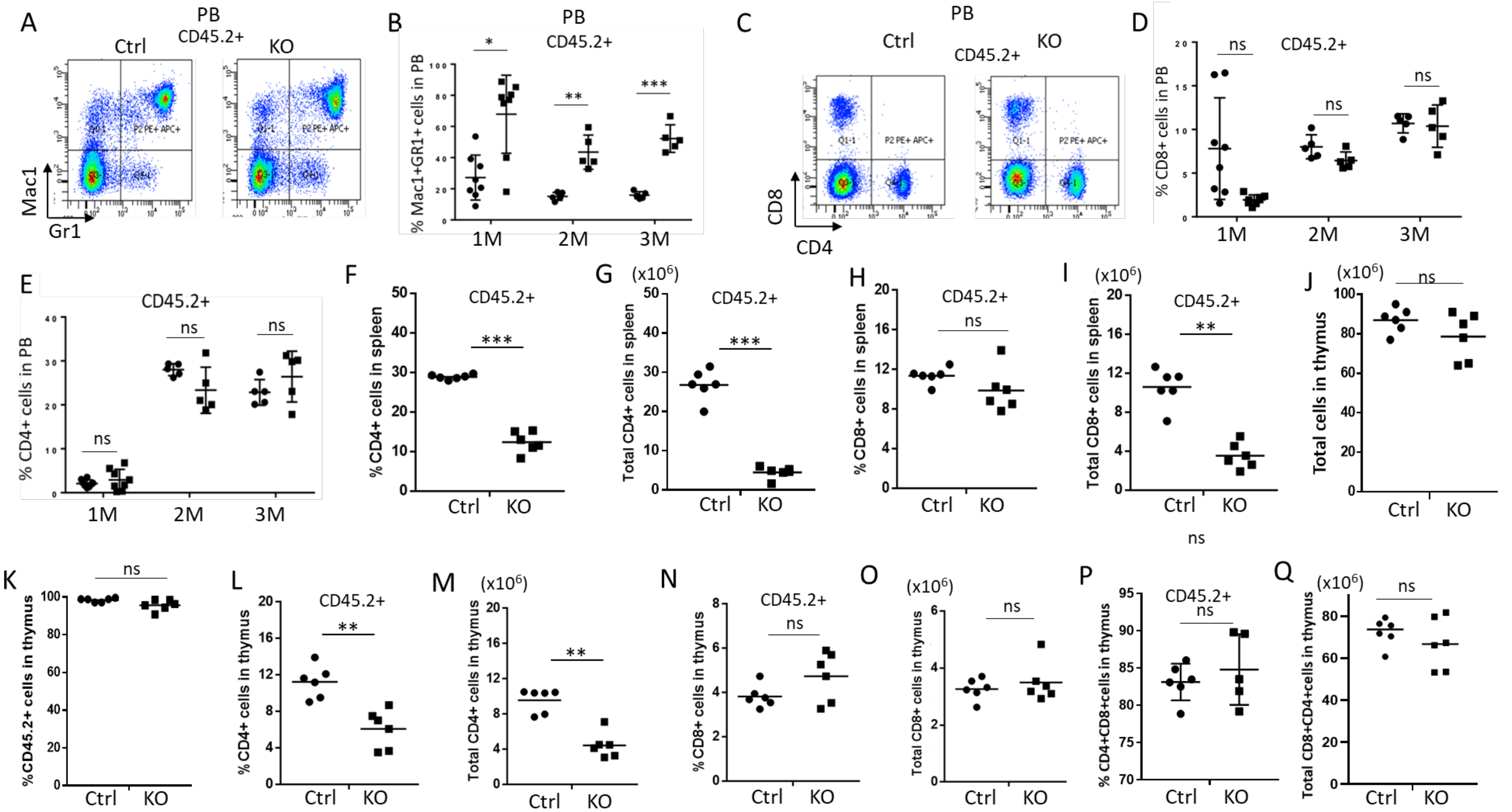

A
PB
CD45.2+
Ctrl
KO
Mac1
Gr1
Q1-1
P2 PE+ APC+
B
PB
CD45.2+
% Mac1+GR1+ cells in PB
1M
2M
3M
*
**
***
C
PB
Ctrl
KO
CD45.2+
CD8
CD4
D
CD45.2+
% CD8+ cells in PB
ns
E
CD45.2+
% CD4+ cells in PB
F
CD45.2+
% CD4+ cells in spleen
G
(x10^6)
Total CD4+ cells in spleen
H
% CD8+ cells in spleen
I
Total CD8+ cells in spleen
J
Total cells in thymus
K
% CD45.2+ cells in thymus
L
% CD4+ cells in thymus
M
Total CD4+ cells in thymus
N
% CD8+ cells in thymus
O
Total CD8+ cells in thymus
P
% CD4+CD8+cells in thymus
Q
Total CD8+CD4+cells in thymus

SI Fig S4

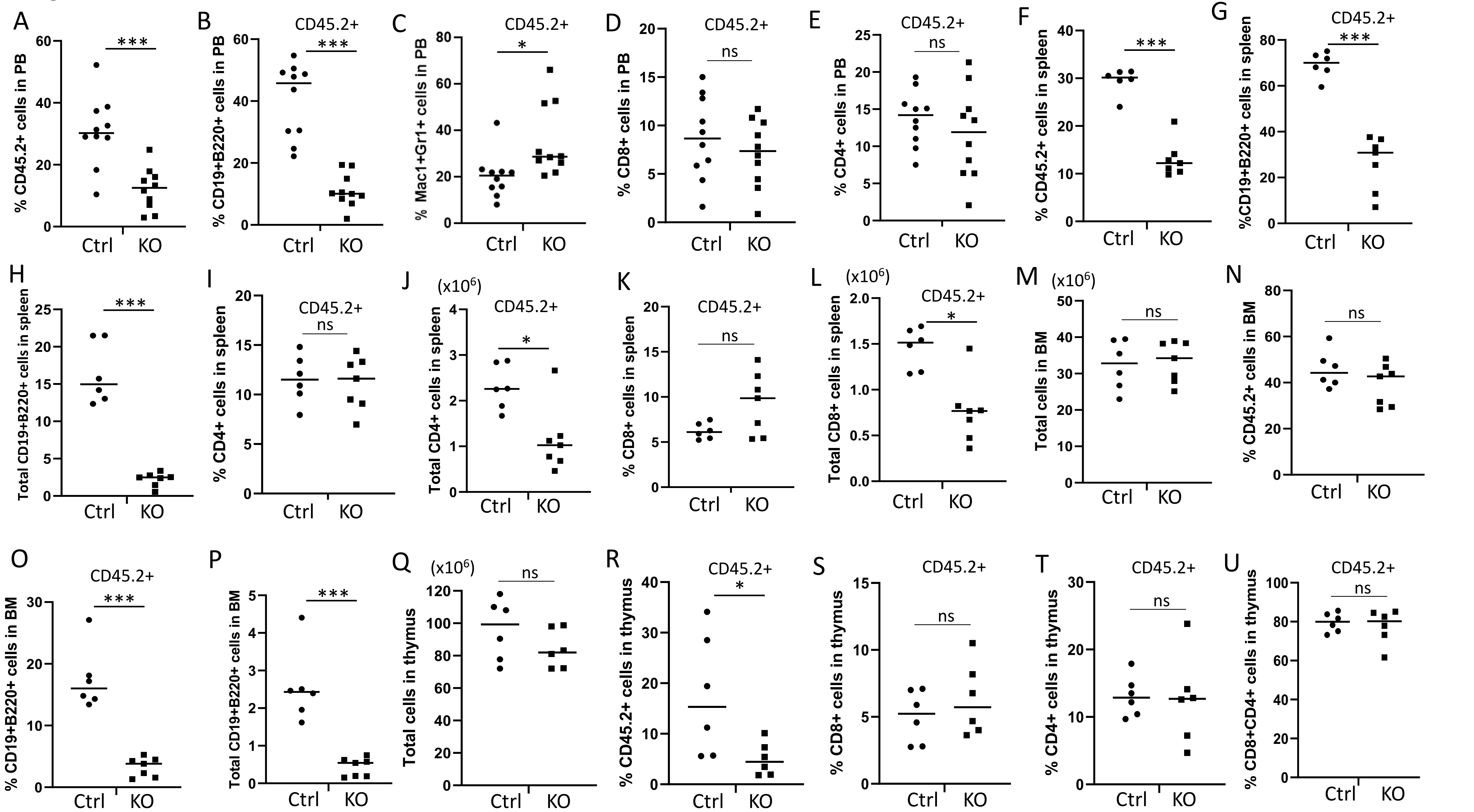

A
% CD45.2+ cells in PB
***
Ctrl
KO
B
CD45.2+
% CD19+B220+ cells in PB
***
Ctrl
KO
C
CD45.2+
% Mac1+Gr1+ cells in PB
*
Ctrl
KO
D
CD45.2+
% CD8+ cells in PB
ns
Ctrl
KO
E
CD45.2+
% CD4+ cells in PB
ns
Ctrl
KO
F
% CD45.2+ cells in spleen
***
Ctrl
KO
G
CD45.2+
%CD19+B220+ cells in spleen
***
Ctrl
KO
H
Total CD19+B220+ cells in spleen
***
Ctrl
KO
I
CD45.2+
% CD4+ cells in spleen
ns
Ctrl
KO
J
(x10⁶)
CD45.2+
Total CD4+ cells in spleen
*
Ctrl
KO
K
CD45.2+
% CD8+ cells in spleen
ns
Ctrl
KO
L
(x10⁶)
CD45.2+
Total CD8+ cells in spleen
*
Ctrl
KO
M
(x10⁶)
Total cells in BM
ns
Ctrl
KO
N
% CD45.2+ cells in BM
ns
Ctrl
KO
O
CD45.2+
% CD19+B220+ cells in BM
***
Ctrl
KO
P
Total CD19+B220+ cells in BM
***
Ctrl
KO
Q
(x10⁶)
Total cells in thymus
ns
Ctrl
KO
R
CD45.2+
% CD45.2+ cells in thymus
*
Ctrl
KO
S
CD45.2+
% CD8+ cells in thymus
ns
Ctrl
KO
T
CD45.2+
% CD4+ cells in thymus
ns
Ctrl
KO
U
CD45.2+
% CD8+CD4+ cells in thymus
ns
Ctrl
KO

SI Fig S5

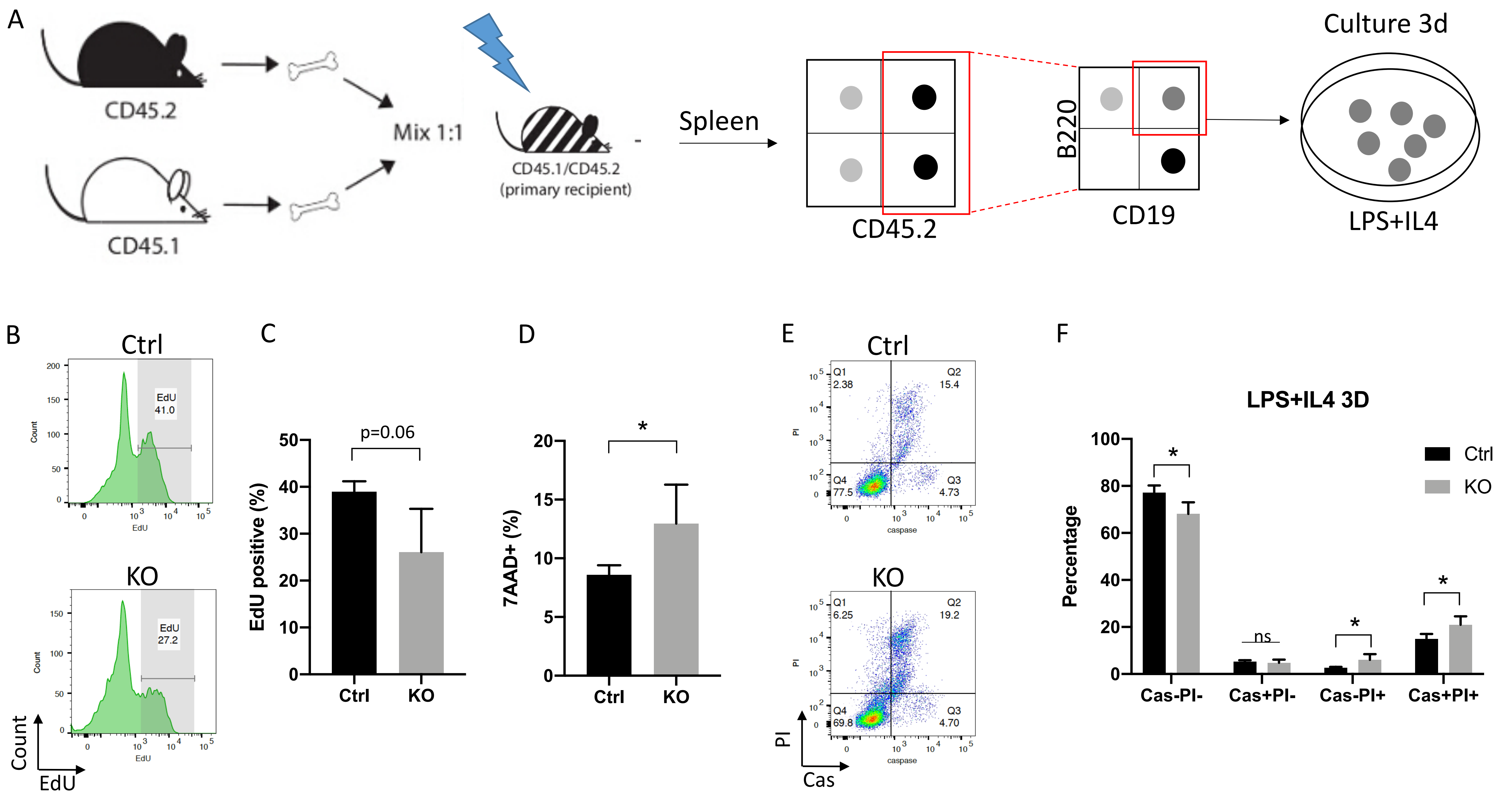
A
CD45.2
CD45.1
Mix 1:1
CD45.1/CD45.2
(primary recipient)
Spleen
CD45.2
B220
CD19
Culture 3d
LPS+IL4
B
Ctrl
EdU
41.0
KO
EdU
27.2
Count
EdU
C
p=0.06
EdU positive (%)
Ctrl
KO
D
*
7AAD+ (%)
Ctrl
KO
E
Ctrl
Q1
2.38
Q2
15.4
Q4
77.5
Q3
4.73
KO
Q1
6.25
Q2
19.2
Q4
69.8
Q3
4.70
PI
Cas
caspase
F
LPS+IL4 3D
Percentage
Ctrl
KO
*
ns
*
*
Cas-PI-
Cas+PI-
Cas-PI+
Cas+PI+

SI Fig S6

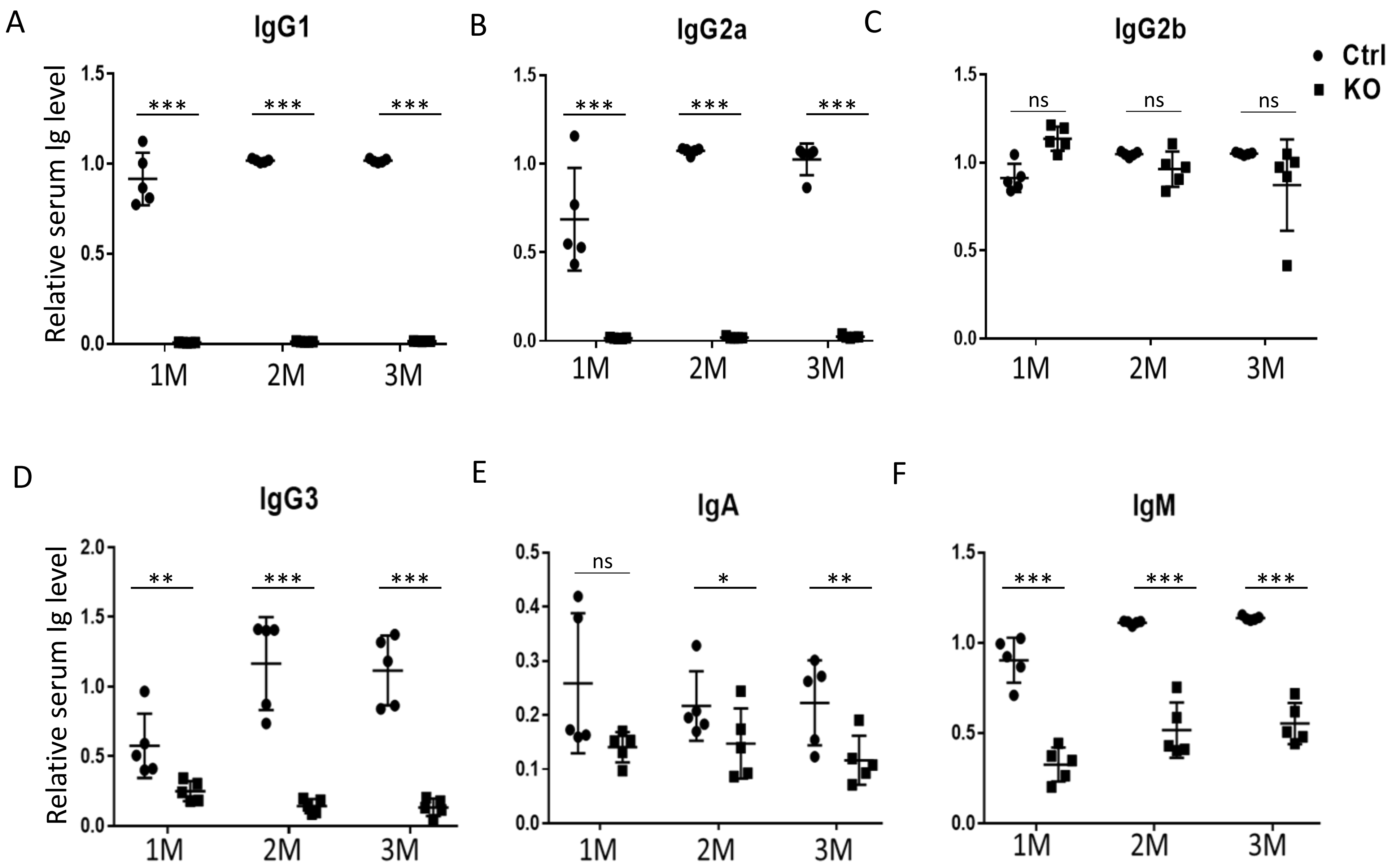

A
IgG1
Relative serum Ig level
0.0
0.5
1.0
1.5
***
***
***
1M
2M
3M
B
IgG2a
0.0
0.5
1.0
1.5
***
***
***
1M
2M
3M
C
IgG2b
0.0
0.5
1.0
1.5
ns
ns
ns
1M
2M
3M
Ctrl
KO
D
IgG3
Relative serum Ig level
0.0
0.5
1.0
1.5
2.0
**
***
***
1M
2M
3M
E
IgA
0.0
0.1
0.2
0.3
0.4
0.5
ns
*
**
1M
2M
3M
F
IgM
0.0
0.5
1.0
1.5
***
***
***
1M
2M
3M

SI Fig S7

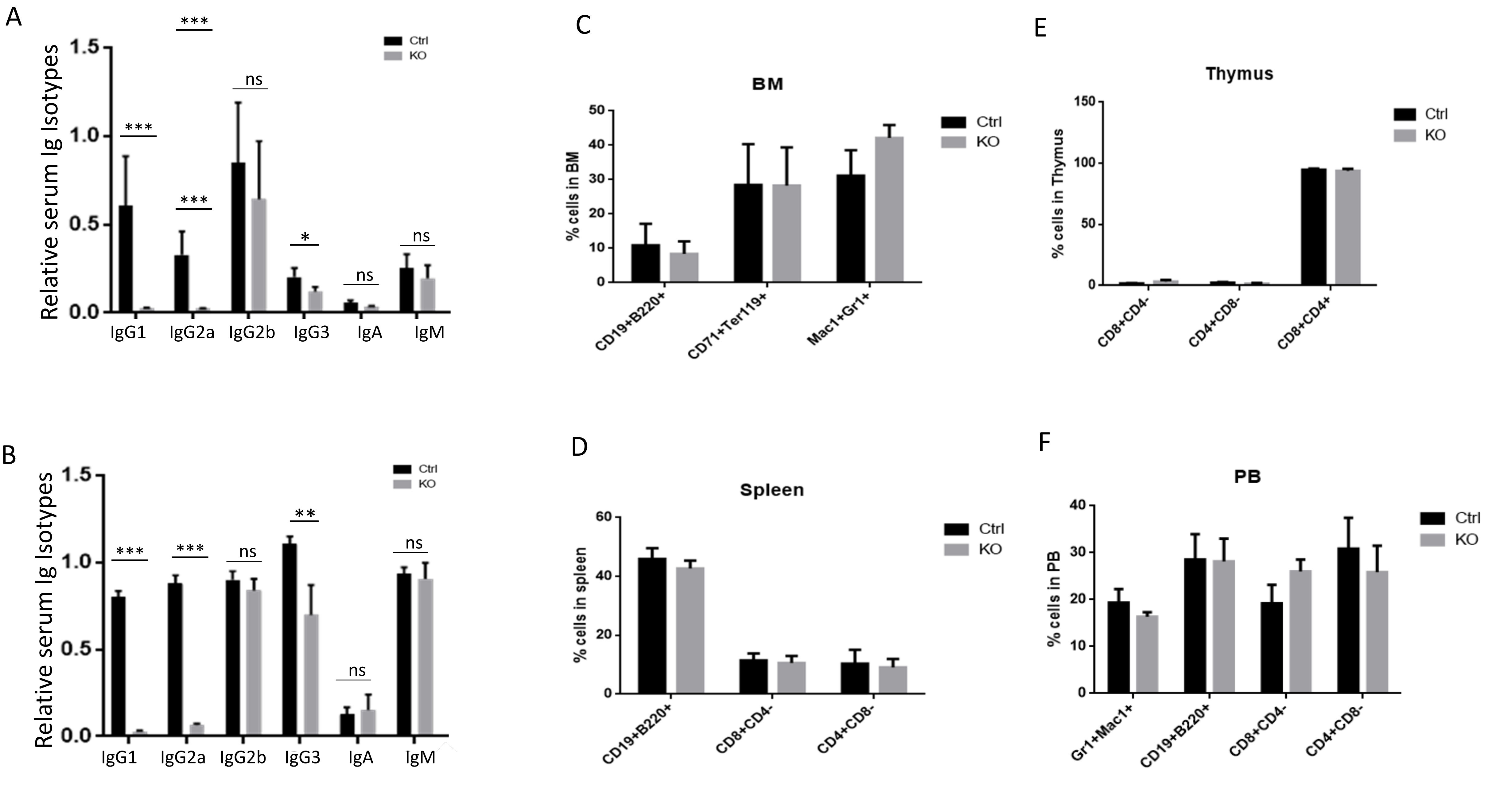
A
Relative serum Ig Isotypes
Ctrl
KO
***
***
***
ns
*
ns
ns
1.5
1.0
0.5
0.0
IgG1
IgG2a
IgG2b
IgG3
IgA
IgM
B
Relative serum Ig Isotypes
Ctrl
KO
***
***
ns
**
ns
ns
1.5
1.0
0.5
0.0
IgG1
IgG2a
IgG2b
IgG3
IgA
IgM
C
BM
% cells in BM
50
40
30
20
10
0
Ctrl
KO
CD19+B220+
CD71+Ter119+
Mac1+Gr1+
D
Spleen
% cells in spleen
60
40
20
0
Ctrl
KO
CD19+B220+
CD8+CD4-
CD4+CD8-
E
Thymus
% cells in Thymus
150
100
50
0
Ctrl
KO
CD8+CD4-
CD4+CD8-
CD8+CD4+
F
PB
% cells in PB
40
30
20
10
0
Ctrl
KO
Gr1+Mac1+
CD19+B220+
CD8+CD4-
CD4+CD8-

SI Fig S8

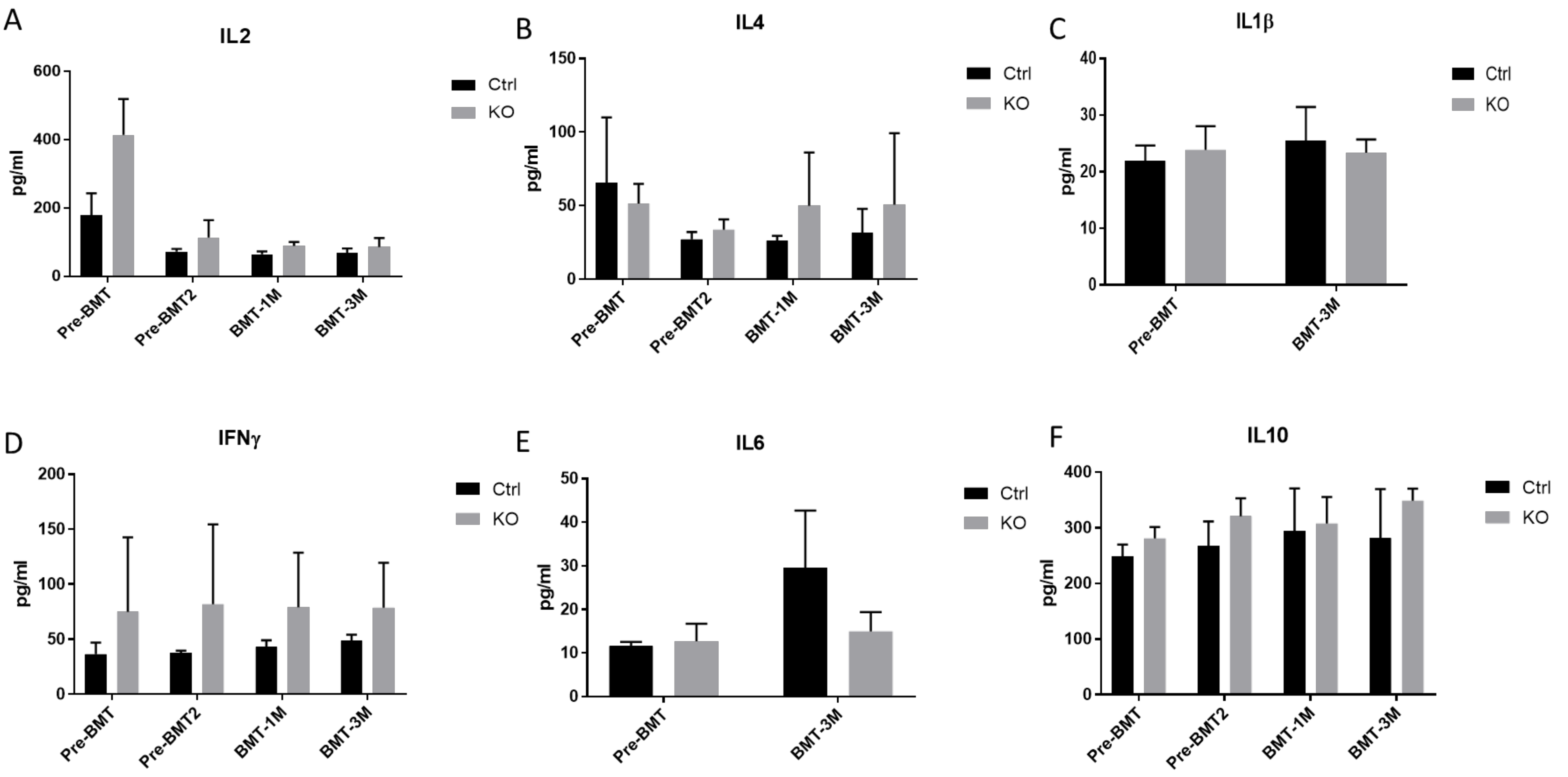
A
IL2
pg/ml
600
400
200
0
Ctrl
KO
Pre-BMT
Pre-BMT2
BMT-1M
BMT-3M
B
IL4
pg/ml
150
100
50
0
Ctrl
KO
Pre-BMT
Pre-BMT2
BMT-1M
BMT-3M
C
IL1β
pg/ml
40
30
20
10
0
Ctrl
KO
Pre-BMT
BMT-3M
D
IFNγ
pg/ml
200
150
100
50
0
Ctrl
KO
Pre-BMT
Pre-BMT2
BMT-1M
BMT-3M
E
IL6
pg/ml
50
40
30
20
10
0
Ctrl
KO
Pre-BMT
BMT-3M
F
IL10
pg/ml
400
300
200
100
0
Ctrl
KO
Pre-BMT
Pre-BMT2
BMT-1M
BMT-3M

SI Fig S9

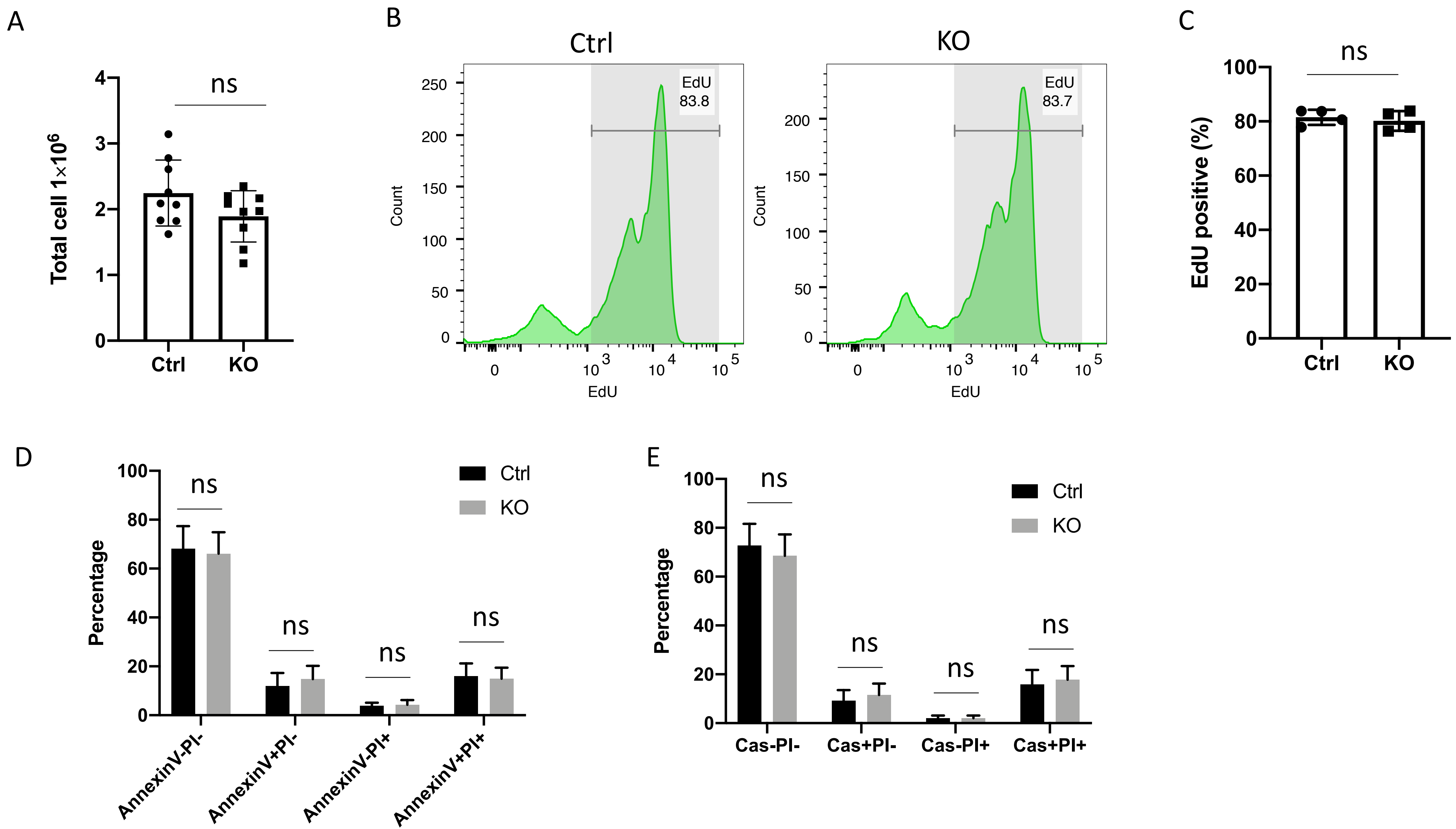
A
ns
Total cell 1×10^6
0
1
2
3
4
Ctrl
KO
B
Ctrl
KO
EdU
83.8
EdU
83.7
Count
EdU
0
50
100
150
200
250
0
10^3
10^4
10^5
C
ns
EdU positive (%)
0
20
40
60
80
100
Ctrl
KO
D
ns
Percentage
Ctrl
KO
AnnexinV-PI-
AnnexinV+PI-
AnnexinV-PI+
AnnexinV+PI+
E
ns
Percentage
Ctrl
KO
Cas-PI-
Cas+PI-
Cas-PI+
Cas+PI+

SI Fig S10

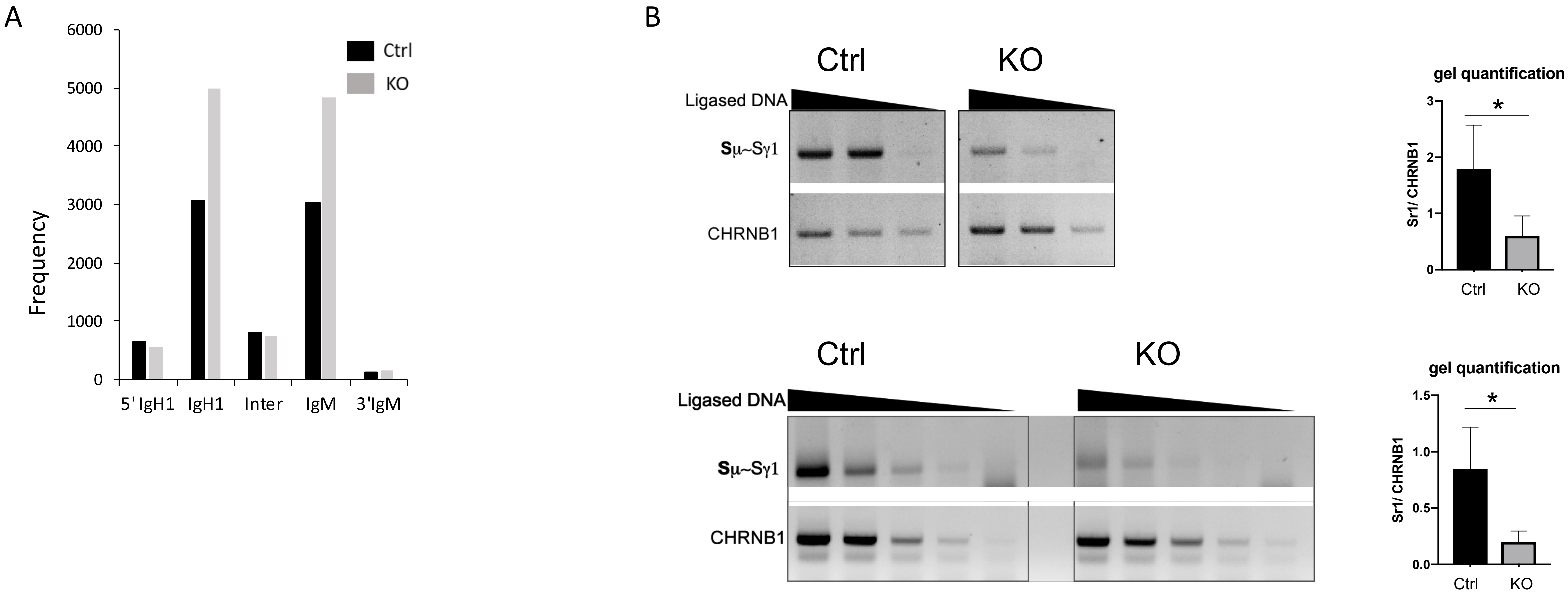
A
6000
5000
4000
3000
2000
1000
0
Frequency
Ctrl
KO
5' IgH1
IgH1
Inter
IgM
3'IgM
B
Ctrl
KO
Ligased DNA
Sμ~Sγ1
CHRNB1
gel quantification
Sr1/ CHRNB1
*
Ctrl
KO
gel quantification
1.5
1.0
0.5
0.0

SI Fig S11

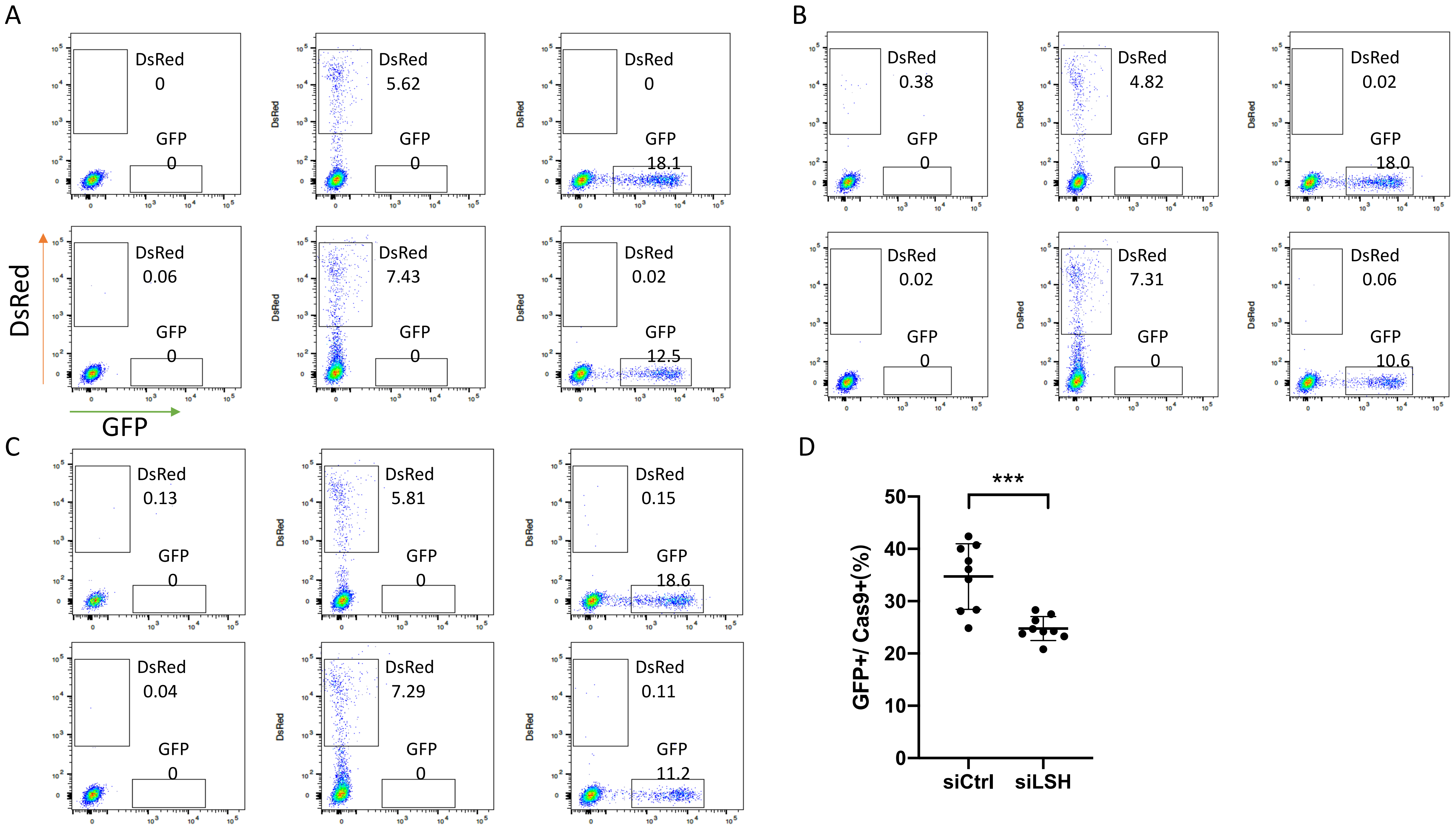
A
DsRed
0
GFP
0
DsRed
5.62
GFP
0
DsRed
0
GFP
18.1
DsRed
0.06
GFP
0
DsRed
7.43
GFP
0
DsRed
0.02
GFP
12.5
DsRed
GFP
B
DsRed
0.38
GFP
0
DsRed
4.82
GFP
0
DsRed
0.02
GFP
18.0
DsRed
0.02
GFP
0
DsRed
7.31
GFP
0
DsRed
0.06
GFP
10.6
C
DsRed
0.13
GFP
0
DsRed
5.81
GFP
0
DsRed
0.15
GFP
18.6
DsRed
0.04
GFP
0
DsRed
7.29
GFP
0
DsRed
0.11
GFP
11.2
D
***
GFP+/ Cas9+(%)
50
40
30
20
10
0
siCtrl
siLSH
